# Minimum energy control for complex networks

**DOI:** 10.1038/s41598-018-21398-7

**Published:** 2018-02-16

**Authors:** Gustav Lindmark, Claudio Altafini

**Affiliations:** Division of Automatic Control, Dept. of Electrical Engineering, Linköping University, SE-58183 Linköping, Sweden

## Abstract

The aim of this paper is to shed light on the problem of controlling a complex network with minimal control energy. We show first that the control energy depends on the time constant of the modes of the network, and that the closer the eigenvalues are to the imaginary axis of the complex plane, the less energy is required for complete controllability. In the limit case of networks having all purely imaginary eigenvalues (e.g. networks of coupled harmonic oscillators), several constructive algorithms for minimum control energy driver node selection are developed. A general heuristic principle valid for any directed network is also proposed: the overall cost of controlling a network is reduced when the controls are concentrated on the nodes with highest ratio of weighted outdegree vs indegree.

## Introduction

Understanding the basic principles that allow to control a complex network is a key prerequisite in order to move from a passive observation of its functioning to the active enforcement of a desired behavior. Such an understanding has grown considerably in recent years. For instance, classical control-theoretical notions like structural controllability have been used to determine a minimal number of driver nodes (i.e., nodes of the network which must be endowed with control authority) that guarantee controllability of a network^1^. Several works have explored the topological properties underlying such notions of controllability^2–7^, or have suggested to use other alternative controllability conditions^8–10^. Several of these approaches are constructive, in the sense that they provide receipts on how to identify a subset of driver nodes that guarantees controllability. However, controllability is intrinsically a yes/no concept that does not take into account the effort needed to control a network^11^. A consequence is that even if a network is controllable with a certain set of driver nodes, the control energy that those nodes require may result unrealistically large. Achieving “controllability in practice” i.e., with a limited control energy, is a more difficult task, little understood in terms of the underlying system dynamics of a network. In addition, in spite of the numerous attempts^9,11–20^, no clear strategy has yet emerged for the related problem of selecting the driver nodes so as to minimize the control energy.

The aim of this paper is to tackle exactly these two issues, namely: i) to shed light on what are the dynamical properties of a network that determine the input energy needed to control a network; and ii) to develop driver node placement strategies requiring minimum control energy.

We show in the paper that for linear dynamics the natural time constants of the modes of the system are key factors in understanding how much energy a control must use. Since the time constants of a linear system are inversely proportional to the real part of its eigenvalues, systems that have eigenvalues near the imaginary axis (i.e., slow dynamics and nearly oscillatory behavior) are easier to control than systems having eigenvalues with large real parts (i.e., fast dynamics, stable or unstable), regardless of the specific metric used to quantify the control energy (in the paper we use Gramian-based measures^17,21^).

The main driver node selection strategy we propose in the paper is based on ranking nodes according to the ratio between weighted outdegree and weighted indegree. This strategy, which is inspired by topological considerations but is still lacking a complete theoretical justification, is very different from choosing a minimum number of driver nodes that guarantee structural controllability. We show that whenever controllability is not an issue (e.g. when the network is strongly connected), such method systematically outperforms a random driver node assignment even by orders of magnitude, in all the considered control metrics and in all considered networks (typically of size ~10^3^ in this paper).

Methods like the one proposed in this paper are based on computing eigenvalues, and hence are necessarily quantitative, i.e., require to assign weights to the edges of a network. However, we show in the paper that our quantitative analysis sheds light on the intrinsic weakness of purely qualitative methods like those based on structural controllability: there are cases in which the Gramian associated to structural controllability is closer to singularity that the one associated with our ranking, regardless of the latter passing or less the structural controllability test. In terms of energy this translates into the following paradox: the control energy of a structurally controllable system can be higher than that of a “structurally uncontrollable” one.

We also show that for the special case of networks of coupled harmonic oscillators, which have purely imaginary spectra, it is possible to obtain explicit criteria for minimum energy driver node placement. Unlike for our main driver node selection strategy, based on weighted outdegree/indegree ratio, which is supported mainly by heuristic topological considerations, the strategies for the special case of purely imaginary spectra rely instead on arguments such as diagonal dominance of the Gramian, and are supported by a fully theoretical analysis. In this case, essentially every Gramian-based controllability measure leads to a tractable minimum energy driver node placement strategy. One of these strategies in particular is exact, and turns out to be very similar to the node ranking mentioned above.

## Methods

### Complete Controllability: merging Reachability and Controllability to 0

A linear system

1 $$\dot{x}=Ax+Bu$$

is controllable if there exists an input *u*(*t*) that transfers the *n*-dimensional state vector *x*(*t*) from any point *x*_*o*_ to any other point *x*_*f*_ in ${{\mathbb{R}}}^{n}$. The Kalman rank condition for controllability, ${\rm{rank}}([B\,AB\,{A}^{2}B\ldots {A}^{k}B])=n$ for *k* sufficiently large, only provides a yes/no answer but does not quantifies what is the cost, in term of input effort, of such state transfer. Once controllability is guaranteed, a possible approach to investigate “controllability in practice” consists in quantifying the least energy that a control requires to accomplish the state transfer, i.e., in minimizing ${\mathcal E} ({t}_{f})={\int }_{0}^{{t}_{f}}\,{\Vert u(\tau )\Vert }^{2}d\tau$ for *u*(*t*) that maps *x*_*o*_ in *x*_*f*_ in a certain time *t*_*f*_. Although in this work we focus on the infinite horizon case (i.e., *t*_*f*_ → ∞), for pedagogical reasons it is instructive to start the analysis from the finite horizon case. In fact, for linear systems like (1), a closed form solution to this problem exists when *t*_*f*_ is finite, and the resulting cost is

2 $$ {\mathcal E} ({t}_{f})={({x}_{f}-{e}^{A{t}_{f}}{x}_{o})}^{T}{W}_{r}^{-1}({t}_{f})\,({x}_{f}-{e}^{A{t}_{f}}{x}_{o}),$$

where the matrix ${W}_{r}({t}_{f})={\int }_{0}^{{t}_{f}}\,{e}^{A\tau }B{B}^{T}{e}^{{A}^{T}\tau }d\tau$ is called the *reachability Gramian*^22^. The control that achieves the state transfer *x*_*o*_ → *x*_*f*_ with minimal cost can be computed explicitly:

3 $$u(t)={B}^{T}{e}^{{A}^{T}({t}_{f}-t)}\,{W}_{r}^{-1}({t}_{f})\,({x}_{f}-{e}^{A{t}_{f}}{x}_{o}),\quad t\in [0,{t}_{f}].$$

Various metrics have been proposed to quantify the difficulty of the state transfer based on the Gramian^21^, like its minimum eigenvalue *λ*_min_(*W*_*r*_), its trace tr(*W*_*r*_), or the trace of its inverse ${\rm{tr}}({W}_{r}^{-1})$, see SI for a more detailed description.

**Figure 1:**
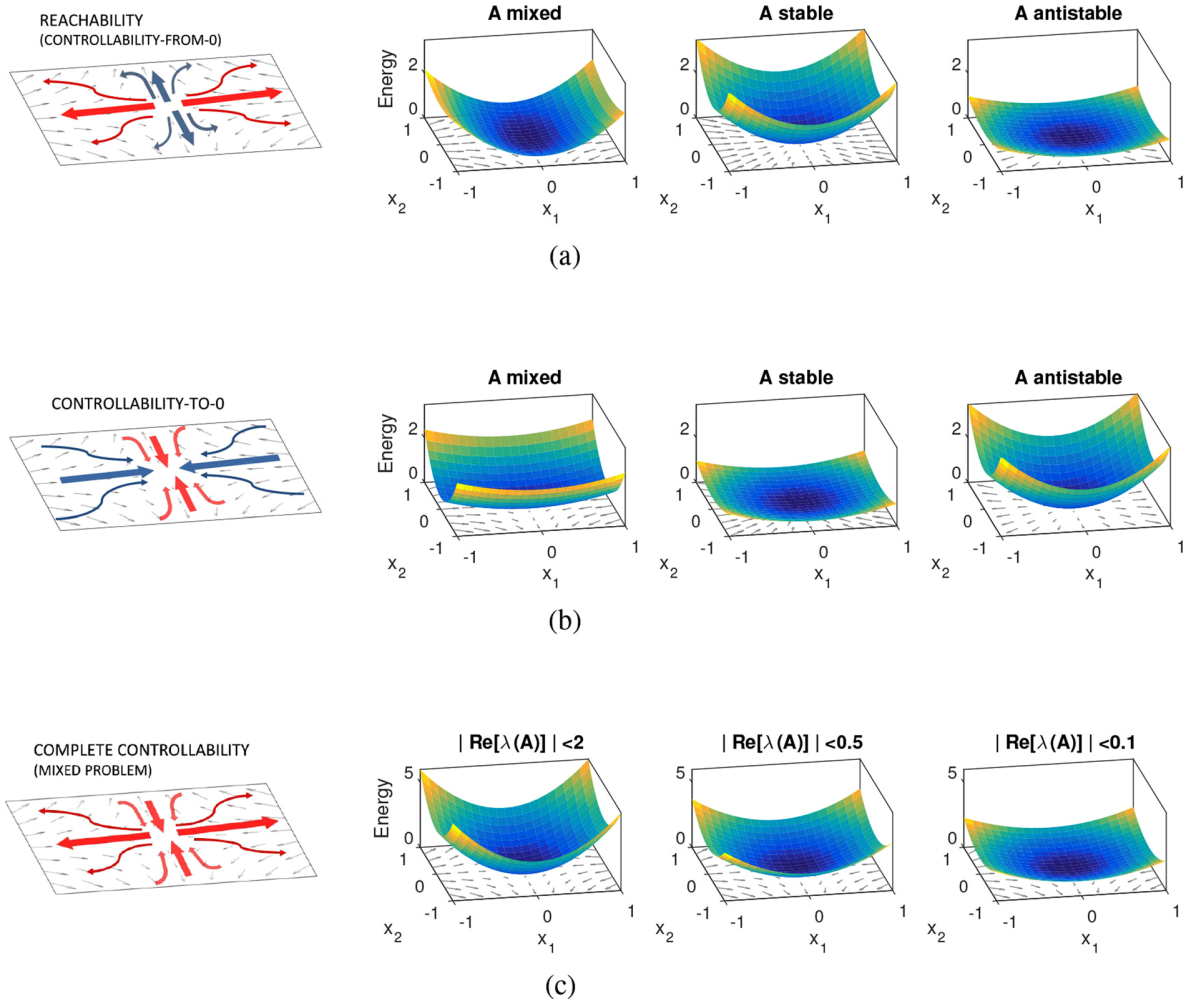
Reachability, Controllability-to-0 and Complete Controllability problems. (**a**) The reachability (or controllability-from-0) problem is difficult along the stable eigendirections of *A* (red curves in the leftmost panel) and easy along the unstable ones (blue). This is reflected in the surfaces of ${ {\mathcal E} }_{r}({t}_{f})={x}_{f}^{T}{W}_{r}^{-1}({t}_{f}){x}_{f}$ shown in the 3 rightmost panels. In particular, the reachability problem requires limited control energy when *A* is antistable (rightmost panel). (**b**) The controllability-to-0 problem is difficult along the unstable eigendirections of *A* (red) and easy along the stable ones (blue). The input energy surfaces, ${ {\mathcal E} }_{c}({t}_{f})={x}_{o}^{T}{W}_{c}^{-1}({t}_{f}){x}_{o}$, reflect these properties. The case of *A* stable requires the least control energy. (**c**) The problem studied in this paper, complete controllability, is a mixture of the two cases, collecting the worst-case situations of both. When the real part of the eigenvalues of *A* is squeezed towards the imaginary axis as in the right panels of Fig. 2(a), the input energy reduces accordingly.

We would like now to describe how (2) depends on the eigenvalues of *A*. In order to do that, one must observe that (2) can be decomposed into contributions originating from two distinct problems: (1): *controllablity*-*from*-*0* (or *reachability*, as it is normally called in control theory^22^) and (2): *controllablity*-*to*-*0*. The first problem consists in choosing *x*_*o*_ = 0, in which case (2) reduces to ${ {\mathcal E} }_{r}({t}_{f})={x}_{f}^{T}{W}_{r}^{-1}({t}_{f}){x}_{f}$, while in the second *x*_*f*_ = 0 leads to ${ {\mathcal E} }_{c}({t}_{f})={x}_{o}^{T}{W}_{c}^{-1}({t}_{f}){x}_{o}$ where ${W}_{c}({t}_{f})={e}^{-{A}^{T}{t}_{f}}{W}_{r}({t}_{f}){e}^{-A{t}_{f}}$ is a second Gramian, called the *controllability Gramian*. The two problems mentioned above, reachability and controllability-to-0, are characterized by different types of difficulties when doing a state transfer, all related to the stability of the eigenvalues of *A*. For instance the reachability problem is difficult along the stable eigendirections of *A* because the control has to win the natural decay of the unforced system to 0, while the unstable eigenvalues help the system escaping from 0 by amplifying any small input on the unstable eigenspaces, see Fig. 1 for a graphical explanation. The surfaces of ${ {\mathcal E} }_{r}({t}_{f})$ shown in Fig. 1(a) reflect these qualitative differences. On the contrary, the influence of the eigenvalues of *A* is the opposite for the controllability-to-0 problem shown in Fig. 1(b). Hence if we want to evaluate the worst-case cost of a transfer between any *x*_*o*_ and any *x*_*f*_ (a problem sometimes referred to as *complete controllability*^23^), we have to combine the difficult cases of the two situations just described.

### Measuring control energy through the infinite-time horizon mixed Gramian

In the complete controllability problem, all the cases leading to a high control energy can be taken into account by combining the two Gramians *W*_*r*_ and *W*_*c*_ into a “mixed” Gramian *W*_*m*_ obtained splitting *A* into its stable and antistable parts and forming a reachability subGramian for the former and a controllability subGramian for the latter^24–26^. Such Gramian can be computed in closed form only when the time of the transfer tends to infinity. In the infinite time horizon, in fact, both *W*_*r*_ and *W*_*c*_ diverge, but their inverses are well-posed and depend only on the stable modes the former and the unstable modes the latter. These are the parts composing the inverse of *W*_*m*_, see Fig. 1(c). Computing *W*_*m*_ and its inverse requires solving jointly two Lyapunov equations. The procedure is explained in detail in the SI. Assume that the spectrum of *A* contains *k*, $0\leqslant k\leqslant n$, eigenvalues with negative real part, and *n* − *k* eigenvalues with positive real part (and no purely imaginary eigenvalues). Then there exist a change of basis *V* bringing *A* into the form:

4 $$[\begin{array}{cc}{\bar{A}}_{1} & 0\\ 0 & {\bar{A}}_{2}\end{array}]=V\,A{V}^{-1}$$

and, correspondingly,

$$[\begin{array}{c}{\bar{B}}_{1}\\ {\bar{B}}_{2}\end{array}]=V\,B$$

with ${\rm{Re}}[\lambda ({\bar{A}}_{1})] < 0$ and ${\rm{Re}}[\lambda ({\bar{A}}_{2})] > 0$. In the new basis, the following two Lyapunov equations hold (see the SI for details on how they are obtained)

5 $$[\begin{array}{cc}{\bar{A}}_{1} & 0\\ 0 & -{\bar{A}}_{2}\end{array}]\,\bar{W}+\bar{W}\,{[\begin{array}{cc}{\bar{A}}_{1} & 0\\ 0 & -{\bar{A}}_{2}\end{array}]}^{T}+[\begin{array}{cc}{\bar{B}}_{1}\,{\bar{B}}_{1}^{T} & 0\\ 0 & {\bar{B}}_{2}\,{\bar{B}}_{2}^{T}\end{array}]=0$$

with

$${\bar{W}}_{m}=[\begin{array}{cc}{\bar{W}}_{\mathrm{1,}r} & 0\\ 0 & {\bar{W}}_{\mathrm{2,}c}\end{array}]$$

the mixed Gramian (the subGramians *W*_1,*r*_ and *W*_2,*c*_ are derived in detail in the SI). Following^24^, the expression of the mixed Gramian in the original basis is ${W}_{m}={V}^{-1}{\bar{W}}_{m}{V}^{-T}$. By construction, the mixed Gramian matrix *W*_*m*_ always exists when *A* has no purely imaginary eigenvalues, and it summarizes the infinite-horizon contribution of the stable eigenvalues to the reachability problem and of the unstable eigenvalues to the controllability to 0 problem (i.e., all cases leading to a high control energy). The three figures of merit *λ*_min_(*W*_*m*_), tr(*W*_*m*_) and ${\rm{tr}}({W}_{m}^{-1})$ are the measures that are mostly used in the paper to quantify the control energy. Although a finite-horizon version of *W*_*m*_ (and ${W}_{m}^{-1}$) can be deduced from the infinite horizon ones (see SI), in this paper we deal exclusively with the infinite horizon case, as often done in the literature^17,22,24^.

### Constructing networks with preassigned spectra

**Figure 2:**
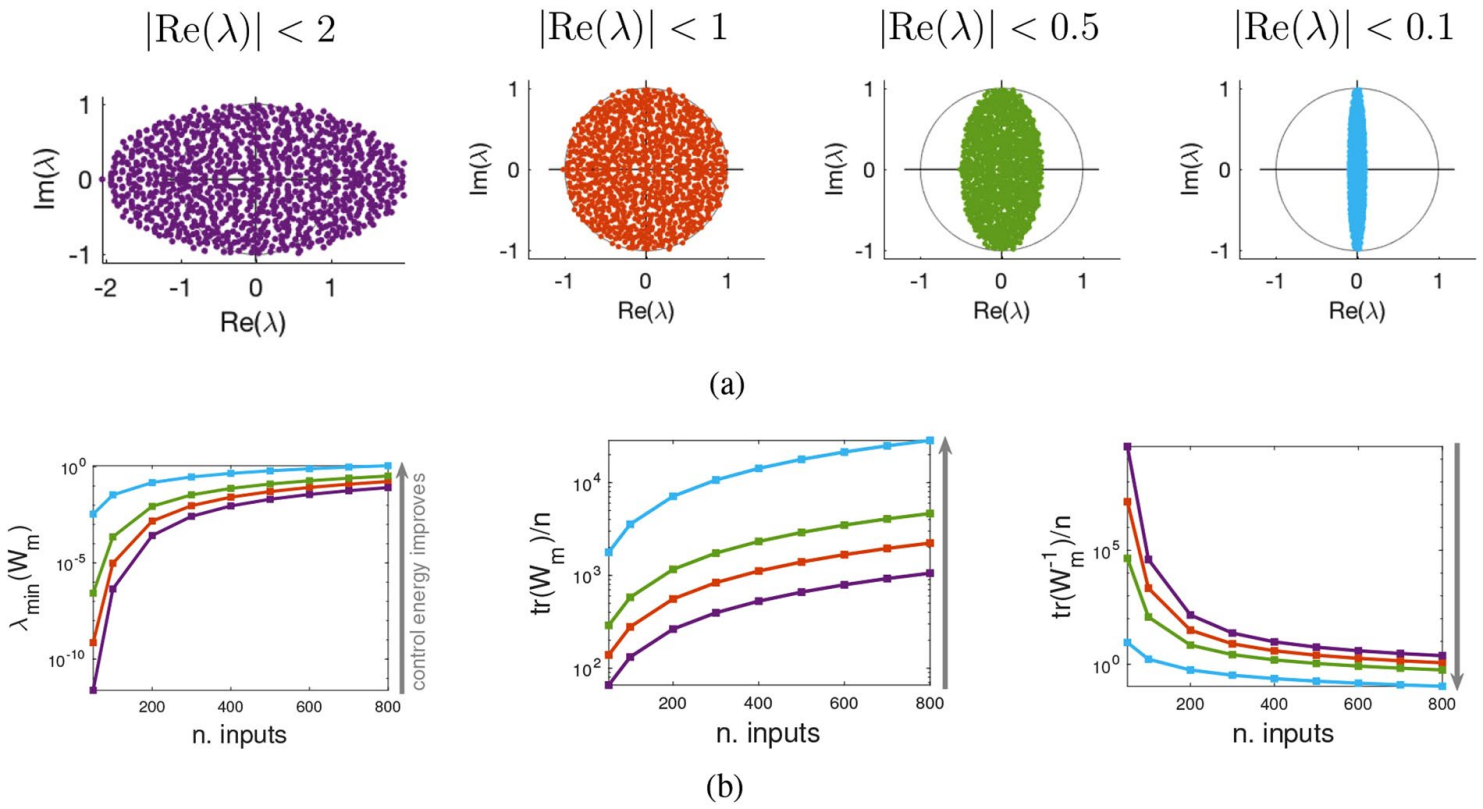
Real part of the eigenvalues and control energy. (**a**) Eigenvalues of a random matrix. The circular/elliptic laws allow to obtain state matrices *A* with eigenvalues in prescribed locations, for instance with predetermined real part. (**b**) Control energy for various metrics when the number of (randomly chosen) inputs grows and Re[*λ*(*A*)] changes. The data show a mean over 100 realizations of dimension *n* = 1000 (for each realization 100 different edge weights assignments are considered). The color code is as in (**a**). Of the three metrics used to measure the control energy, *λ*_min_(*W*_*m*_) (left) and tr(*W*_*m*_) (middle) should both be maximized, while ${\rm{tr}}({W}_{m}^{-1})$ (right) should be minimized in order to improve the control energy (see direction of the arrow on the left of each panel). For all three metrics, the performances are strictly a function of the position of the eigenvalues of *A*. The minimum of the control energy is achieved when the eigenvalues have very small real part (cyan) and worsen with growing real part, following the order: cyan, green, red, violet.

The circular and elliptic laws^27^ are used in the paper to construct random networks with eigenvalues in desired regions. The circular law states that a matrix *A* of entries ${a}_{ij}/\sqrt{n}$ where *a*_*ij*_ are i.i.d. random variables with zero-mean and unit variance has spectral distribution which converges to the uniform distribution on the unit disk as *n* → ∞, regardless of the probability distribution from which the *a*_*ij*_ are drawn. A numerical example is shown in Fig. 2(a) (second panel from left). A random matrix is typically a full matrix, meaning that the underlying graph of interactions is fully connected. The circular law is however valid also for sparse matrices, for instance for Erdös-Rényi (ER) topologies. If *p* is the edge probability, then $A=({a}_{ij})/\sqrt{p\cdot n}$ still has eigenvalues distributed uniformly on the unit disk, see Fig. S1(a).

A generalization of the circular law is the *elliptic law*, in which the unit disk containing the eigenvalues of *A* is squeezed in one of the two axes. To do so, the pairs of entries {*a*_*ij*_, *a*_*ji*_} of *A* have to be drawn from a bivariate distribution with zero marginal means and covariance matrix expressing the compression of one of the two axes. Various examples of elliptic laws are shown in the panels of Fig. 2(a). Also elliptic laws generalize to sparse matrices, see Fig. S1(a).

## Results

### Control energy as a function of the real part of the eigenvalues of *A*

In a driver node placement problem, the inputs affect a single node, hence the columns of *B* are always elementary vectors, i.e., vectors having one entry equal to 1 and the rest equal to 0. When *A* is a random matrix, the underlying graph is generically fully connected, hence issues like selection of the minimal number of driver nodes based on the connectivity of the graph become irrelevant. In this work we always consider cases in which the driver nodes we have available are enough to guarantee controllability. Having disentangled the problem from topological aspects, the dependence of the control energy from other factors, like the spectrum of *A*, becomes more evident and easier to investigate. If for instance we place driver nodes at random and use the infinite-horizon mixed Gramian *W*_*m*_ to form the various energy measures mentioned above for quantifying the control energy, then we have the results shown in Fig. 2(b). As expected, all indicators improve with the number of inputs, because the control effort gets spread over more nodes. What is more interesting is that when we repeat the computation for the various spectral distributions of Fig. 2(a), the result is that the control energy decreases when the (absolute value of the) real part of the eigenvalues of *A* decreases. All measures give an unanimous answer on this dependence, regardless of the number of inputs considered. In particular, when *A* has eigenvalues which are very close to the imaginary axis (rightmost panel of Fig. 2(a) and cyan curves in Fig. 2(b)) then *λ*_min_(*W*_*m*_) is bigger (i.e., the worst-case controllability direction is easiest to control), but also tr(*W*_*m*_) increases and ${\rm{tr}}({W}_{m}^{-1})$ decreases (meaning, in both cases, that the average energy needed for controllability on all directions decreases).

An identical result is valid also for sparse matrices. In particular, for ER graphs with edge probability *p* = 0.05 and coefficients from a bivariate normal distribution (yielding elliptic laws as in Fig. S1(b)), the various norms used to quantify the control energy are shown in Fig. S1(b). Their pattern is identical to the full graph case of Fig. 2(b).

The computations shown in Fig. 2(b) are performed with the infinite-horizon mixed Gramian *W*_*m*_ described in the SI, because such *W*_*m*_ can be easily computed in closed form. A finite-horizon *W*_*m*_(*t*_*f*_) can be approximately obtained from it, but the arbitrarity of *t*_*f*_ makes it hard to set up an unbiased comparison of the various spectral distributions of *A* of Fig. 2(a), which are characterized by widely different time constants (inversely correlated to the amplitude of the real part of *λ*(*A*)). Observe in Fig. S2 how the various measures of controllability computed with a finite-time *W*_*m*_(*t*_*f*_) tend all to the infinite-time *W*_*m*_ but with different speeds.

### Driver node placement based on weighted connectivity

**Figure 3:**
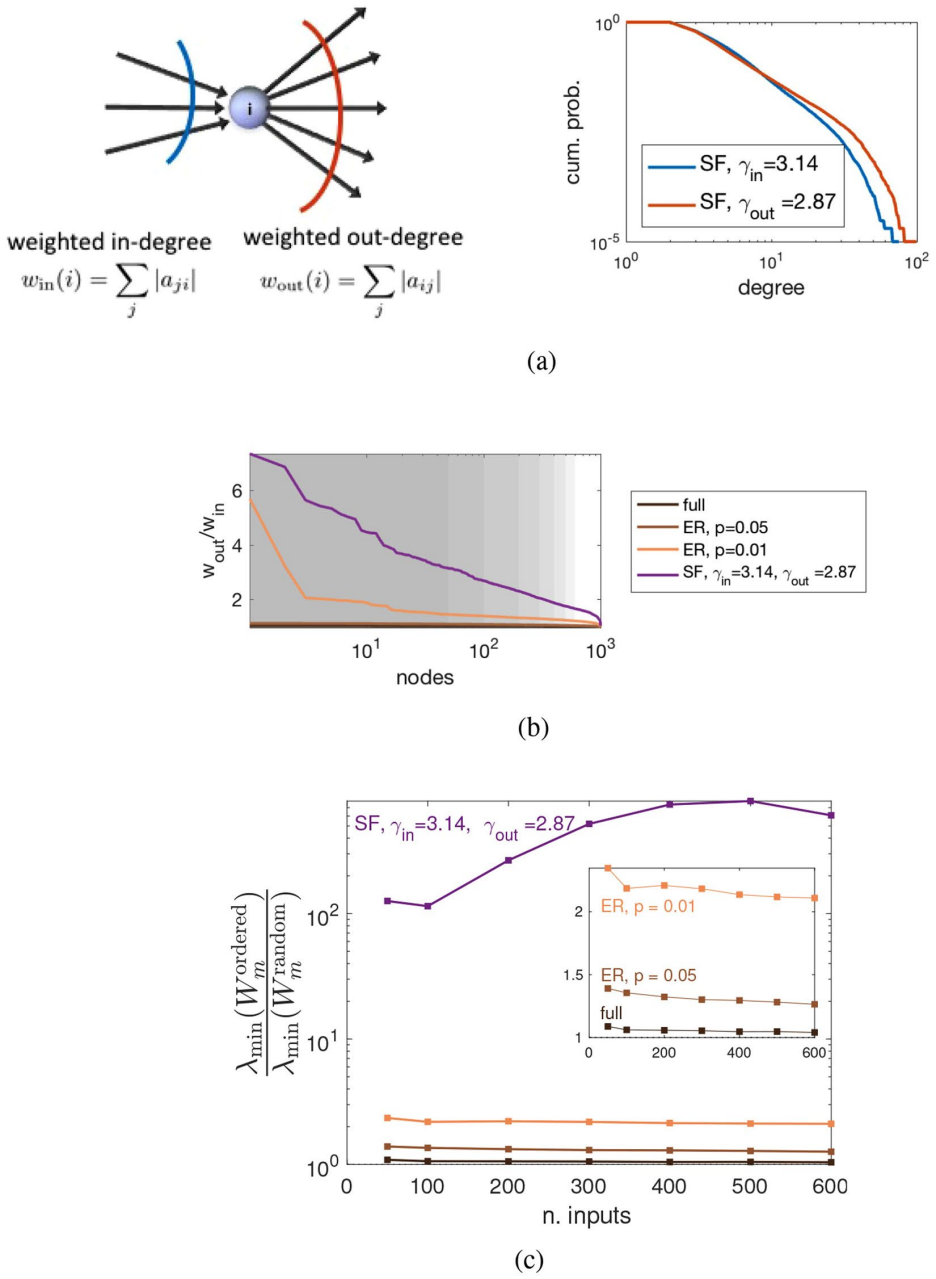
Driver node placement strategy: ranking according to *r*_*w*_ = *w*_out_/*w*_in_. (**a**) Interpretation of the ranking strategy (left panel). The selection of driver nodes starts from those having the highest ratio *r*_*w*_, meaning those that are dominated by (weighted) out-degree. Networks that are SF directed graphs with indegree exponent bigger than out-degree exponent (in the right panel *γ*_in_ = 3.14 and *γ*_out_ = 2.87) have a large fraction of nodes with this characteristic. (**b**) The ratio *r*_*w*_ of ranked nodes is shown for ER networks of different densities and for the SF network shown in (**a**). Indeed for SF the fraction of nodes having high *r*_*w*_ is much bigger than that of ER networks. The shaded areas represent the values of *m* used in our computations of control energy. (**c**) Comparison between control energy (here measured according to *λ*_min_(*W*_*m*_)) for the *r*_*w*_-ranking (denoted ${\lambda }_{{\rm{\min }}}({W}_{m}^{{\rm{ordered}}})$) and random driver node selection (denoted ${\lambda }_{{\rm{\min }}}({W}_{m}^{{\rm{random}}})$). The ratio between the two quantities is shown for the ER and SF networks mentioned in (**a**,**b**). For ER networks, the improvement in control energy increases with the sparsity of the graph (inset: zoomed comparison in linear scale). For the SF networks, the improvement is remarkably more significant (two orders of magnitude, violet curve, see also Fig. S5 for more details). Measures are means over 100 realizations of size *n* = 1000; for each realization 100 edge weight assignments are tested.

In the analysis carried out so far the driver nodes were chosen randomly. A topic that has raised a remarkable interest in recent times (and which is still open in our knowledge) is devising driver node placement strategies that are efficient in terms of input energy^12–17,19,20^. If we consider as weighted indegree and outdegree at node *i* the sum of the weights in absolute value of all incoming or outgoing edges, i.e., ${w}_{{\rm{in}}}(i)={\sum }_{j=1}^{n}\,|{a}_{ij}|$ and ${w}_{{\rm{out}}}(i)={\sum }_{j=1}^{n}\,|{a}_{ji}|$ (a normalization factor such as $\sqrt{p\cdot n}$ can be neglected), see Fig. 3(a), then a strategy that systematically beats random input assignment consists in ranking the nodes according to the ratio *r*_*w*_(*i*) = *w*_out_(*i*)/*w*_in_(*i*) and placing inputs on the nodes with highest *r*_*w*_. In Fig. 3(c) the *λ*_min_(*W*_*m*_) of this driver node placement strategy is compared with a random driver node selection. If for full graphs the improvement is minimal, as the graphs become sparser it increases, and for ER networks with *p* = 0.01 the *λ*_min_(*W*_*m*_) obtained by controlling nodes with high *r*_*w*_ is more than twice that of the random choice of controls, see Fig. 3(b). As can be seen in Fig. S3, all measures of input energy show a qualitatively similar improvement. The topology of the network can be used to render the values of *r*_*w*_ more extreme, for instance choosing direct scale-free (SF) graphs with indegree exponent bigger than outdegree exponent^28^ (and at the same time guaranteeing strong connectivity of the ensuing graphs, in order to avoid problems with minimal controllability), see Fig. 3(a,b). For these skewed SF networks, the improvement in choosing driver nodes with high *r*_*w*_ becomes much more substantial, even of orders of magnitude bigger than a random selection, see Figs 3(c), S4 and S5 for more details.

### Application to real-world networks

**Figure 4:**
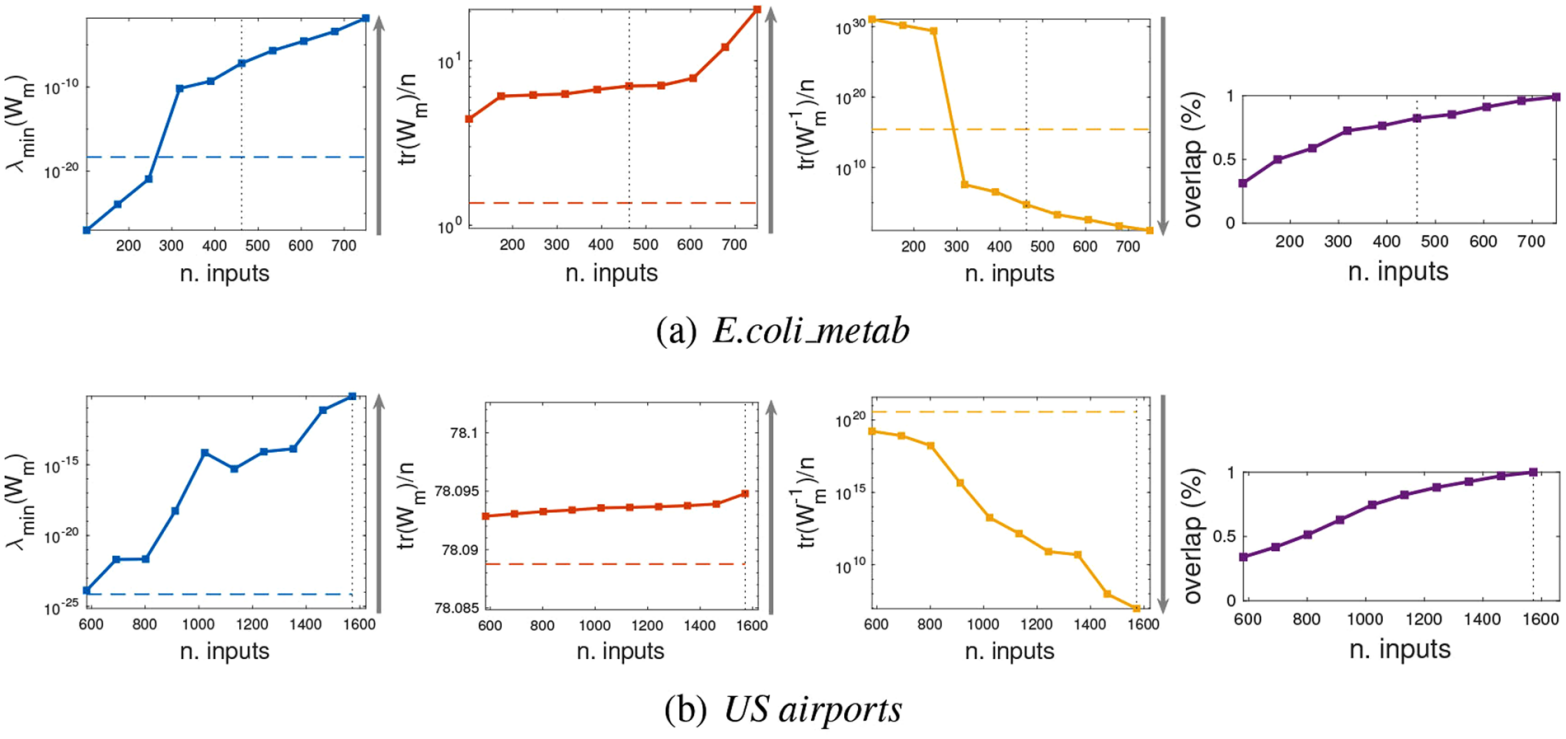
Driver node placement according to *r*_*w*_ = *w*_out_/*w*_in_ for two real-world networks. (**a**) *Ecoli*-*metabol*. (**b**) *US Airports*. In both cases the control energy measures *λ*_min_(*W*_*m*_), tr(*W*_*m*_), and ${\rm{tr}}({W}_{z}^{-1})$ are shown in the 3 leftmost panels (solid lines), when the number *m*_*e*_ of driver nodes (chosen according to *r*_*w*_) grows. The horizontal dashed line is the control energy in correspondence of the *m*_*c*_ driver nodes required by structural controllability. The vertical dotted line is the value of *m*_*e*_ at which the *r*_*w*_-ranked nodes achieve structural controllability (it is *m*_*e*_ = *n* in *US Airports*). The rightmost panel shows how many of the *m*_*e*_ *r*_*w*_-ranked nodes overlap with the *m*_*c*_ nodes of structural controllability.

In order to verify whether the proposed driver node placement strategy is effective also on more realistic data, we tested our algorithm on several networks collected from the literature, and representing complex systems of biological (transcriptional, metabolic and signaling), ecological (food-web), social, transportation and trade type, see Table 1. As for most real datasets, the underlying graphs are not strongly connected, hence controllability is not automatically verified. In order to guarantee it, it is necessary to assign a certain number *m*_*c*_ of driver nodes, for instance chosen according to structural controllability and computed using a maximum matching algorithm^1^, see Table 1. If edge weights are assigned (here drawn from a uniform distribution), then in correspondence of such minimum number of driver nodes it is possible to estimate the control energy via the three measures *λ*_min_(*W*_*m*_), tr(*W*_*m*_), and ${\rm{tr}}({W}_{m}^{-1})$, see Figs 4, S7 and S8. When instead the driver nodes are chosen according to our ranking strategy *r*_*w*_, then there is no guarantee of achieving controllability in a structural sense. In theory, lack of structural controllability corresponds to a singular Gramian, but in practice it corresponds to a poorly conditioned Gramian, with *λ*_min_(*W*_*m*_) small and ${\rm{tr}}({W}_{m}^{-1})$ big. One expects then a higher control energy than when structural controllability is guaranteed. This in fact happens in some of the networks we are considering. For instance in the *E*. *coli*-*metab* network of Fig. 4(a), indeed when *m*_*e*_ nodes are chosen according to *r*_*w*_, then if *m*_*e*_ = *m*_*c*_ two of the three control energy measures (*λ*_min_(*W*_*m*_) and ${\rm{tr}}({W}_{m}^{-1})$) are worst than in the structurally controllable case. However, for other networks, such as the *US Airports* network of Fig. 4(b), when *m*_*e*_ = *m*_*c*_ the control energy for driver nodes chosen according to *r*_*w*_ is already better than for structural controllability, and it improves further when *m*_*e*_ > *m*_*c*_. A similar behavior is observed in several of the networks analyzed, see Figs S7 and S8. In all networks, the control energy improves as *m*_*e*_ increases (as expected), and it becomes better than that of the *m*_*c*_ driver nodes required by structural controllability before the *r*_*w*_-ranked nodes begin to fulfill the structural controllability test. Notice how in nearly all networks tr(*W*_*m*_) (which is less sensitive to singularity of *W*_*m*_) improves when we choose *r*_*w*_-ranked driver nodes.

**Table 1:** Various networks used in this study.

Network	nodes	edges	*m*_*c*_	*m*_*f*_	source (ref. n.)
**Biological, transcriptional**					
*E*. *coli*	1623	3515	1472	75	^38^
Yeast	664	1066	550	57	^39^
**Biological, metabolic**					
*E*. *coli*	757	6116	102	327	^40^
Yeast	780	4421	142	319	^41^
**Biological, signalling**					
Macrophage	660	1549	179	240	^42^
Toll-like	672	2194	144	264	^43^
**Food-web**					
Florida	128	2106	30	49	^44^
Mangdry	97	1491	22	37	^44^
**Social**					
Moreno highschool	70	366	3	33	^45^
Advogato	5042	47067	1194	1924	^46^
**Transport and Trade**					
US Airport	1572	28235	582	495	^47^
Wheat	166	1782	35	65	^48^

*m*_*c*_ represents the number of driver nodes needed to guarantee structural controllability. *m*_*f*_ the number of extra driver nodes (in addition the previous *m*_*c*_) selected to reduce the control energy.

**Figure 5:**
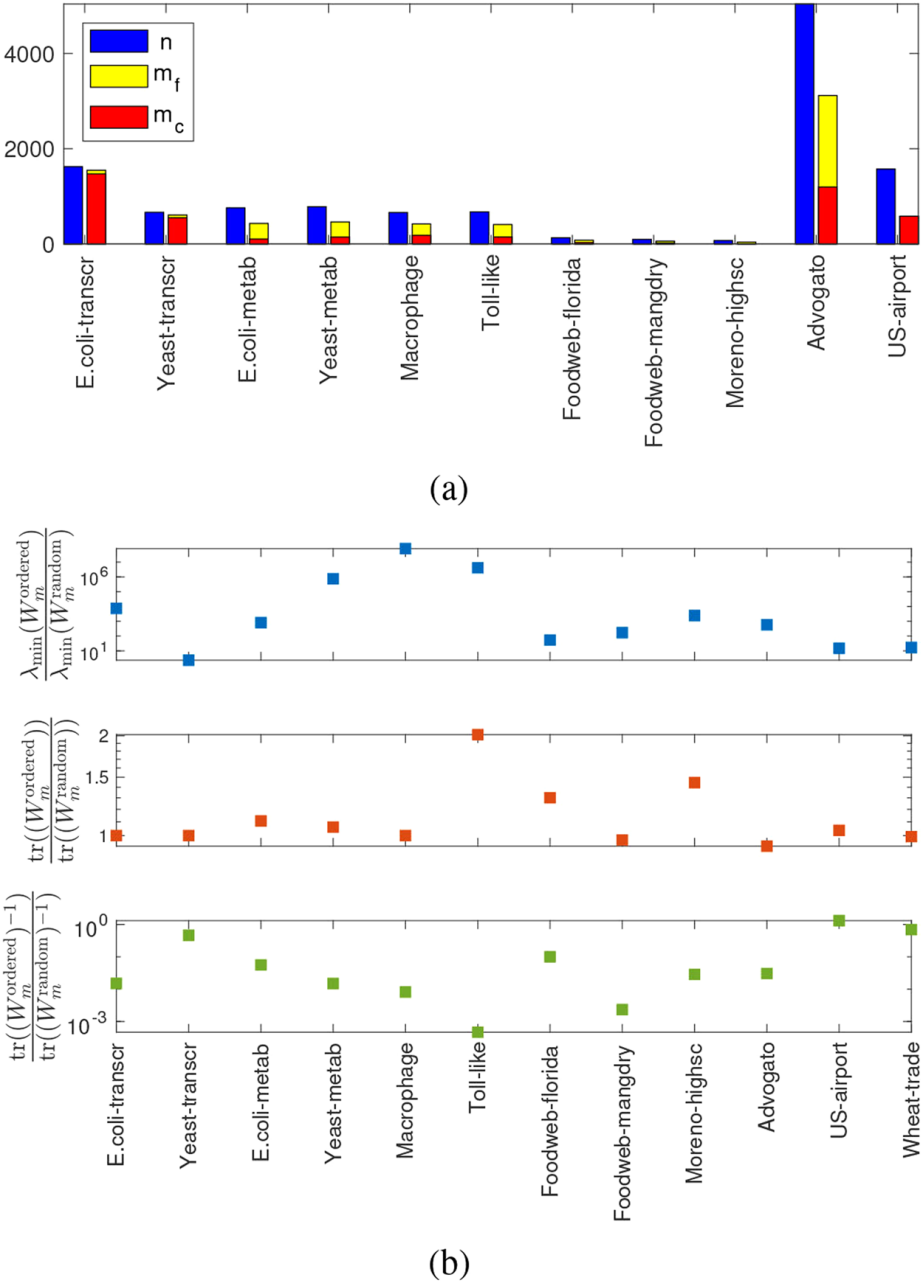
Control energy of driver node placement according to *r*_*w*_ = *w*_out_/*w*_in_ for the real-world networks of Table 1. (**a**) The real-world networks used. The number of nodes (*n*) is shown in blue, the minimal number of driver nodes needed to achieve controllability (*m*_*c*_) is shown in red, and the number of additional driver nodes selected (*m*_*f*_) in yellow. See also Table 1. For some networks, like for the two transcriptional networks, *m*_*c*_ amounts to a large fraction of *n*. (**b**) Comparison of control energies between a choice of *m*_*f*_ nodes according to *r*_*w*_ and a random choice. The two choices give rise to two different mixed Gramians, denoted respectively ${W}_{m}^{{\rm{ordered}}}$ and ${W}_{m}^{{\rm{random}}}$. These Gramians are used to form the three measures of control energy *λ*_min_(*W*_*m*_), tr(*W*_*m*_), and ${\rm{tr}}({W}_{m}^{-1})$. The ratios between the two resulting values for these three metrics are shown in the three panels for the real networks considered in this study. In basically all cases the control energy improves (i.e., $\tfrac{{\lambda }_{{\rm{\min }}}({W}_{m}^{{\rm{ordered}}})}{{\lambda }_{{\rm{\min }}}({W}_{m}^{{\rm{random}}})}\gg 1$, $\tfrac{{\rm{tr}}(({W}_{m}^{{\rm{ordered}}}))}{{\rm{tr}}(({W}_{m}^{{\rm{random}}}))} > 1$, and $\tfrac{{\rm{tr}}(({W}_{m}^{{\rm{ordered}}}{)}^{-1})}{{\rm{tr}}(({W}_{m}^{{\rm{random}}}{)}^{-1})}\ll 1$), often by several orders of magnitude, even when *m*_*f*_ is small.

An alternative test that can be carried out to evaluate the effect of our ranking strategy on the control energy consists in choosing the *m*_*c*_ driven nodes prescribed by structural controllability, plus extra *m*_*f*_ nodes according to the ranking *r*_*w*_. Here we have decided to select a number of extra driver nodes equal to *m*_*f*_ = (*n* − *m*_*c*_)/2 (i.e., half of the remaining nodes), in two different ways: i) according to the ratio *r*_*w*_, and ii) randomly. The resulting numbers for our networks are given in Fig. 5(a) and in Table 1. The results in terms of control energy are illustrated in Fig. 5(b). For all networks, all three metrics *λ*_min_(*W*_*m*_), tr(*W*_*m*_), and ${\rm{tr}}({W}_{m}^{-1})$ are essentially always confirming that the driver node selection strategy based on *r*_*w*_ is outperforming significantly a random node assignment. In particular, for the two nonlinear metrics (*λ*_min_(*W*_*m*_) and ${\rm{tr}}({W}_{m}^{-1})$), the improvement is often of several orders of magnitude, i.e., even better than in the skewed SF networks discussed in Fig. 3.

### Systems with purely imaginary eigenvalues: the case of coupled harmonic oscillators

From what we have seen above, the control energy is least when the real part of the eigenvalues tends to vanish. In the extreme case of purely imaginary eigenvalues, it is possible to obtain explicit driver node placement criteria that minimize the control energy. A special case of linear system with purely imaginary eigenvalues is a network of *n* coupled harmonic oscillators, represented by a system of second order ODEs

6 $$M\ddot{q}+Kq=Bu$$

where *M* = *M*^*T*^ > 0 is the inertia matrix, $K={K}^{T}\geqslant 0$ is the stiffness matrix, typically of the form *K* = *K*_*d*_ + *L*, with ${K}_{d}\geqslant 0$ diagonal and *L* a Laplacian matrix representing the couplings. In (6) the controls are forces, and the columns of the input matrix *B* are elementary vectors in correspondence of the driver nodes. The state space representation of (6) is

7 $$\dot{x}={A}_{o}x+{B}_{o}u$$

with

$$x=[\begin{array}{c}Mq\\ M\dot{q}\end{array}]\in {{\mathbb{R}}}^{2n},\quad {A}_{o}=[\frac{0}{-K\,{M}^{-1}}|\frac{I}{0}],\quad {\rm{and}}\quad {B}_{o}=[\frac{0}{B}].$$

**Figure 6:**
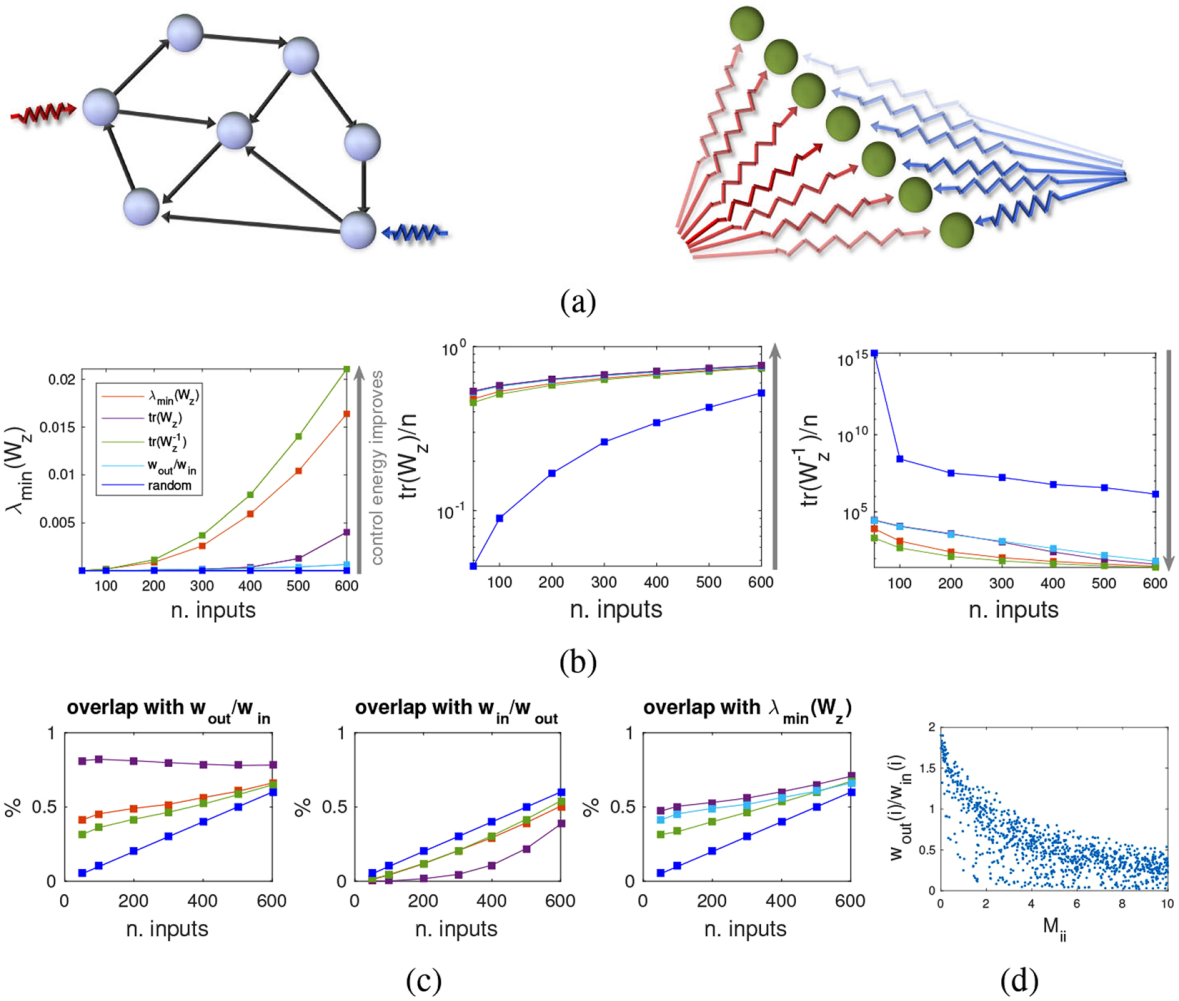
Driver node placement strategies for a network of coupled harmonic oscillators. (**a**) The concept of driver node is basis dependent: when the basis changes in state space (for instance we pass from (7) to (8)), the control inputs no longer target a single node, but become spread across the entire state space (now decoupled into non-interacting modes). (**b**) Comparison of different driver node placement strategies for *n* = 1000 coupled harmonic oscillators. Shown are means over 100 realizations (with 100 edge weights samples taken for each realization). The diagonal Gramian *W*_*z*_ of (9) is used to quantify the control energy. Red: driver node placement based on *λ*_min_(*W*_*z*_). Violet: placement based on tr(*W*_*z*_). Green: placement based on ${\rm{tr}}({W}_{z}^{-1})$. Cyan: placement based on *w*_out_/*w*_in_. Blue: random input assignment. All driver node placement strategies always beat a random assignment, often by orders of magnitude. The green and red curves give similar performances and so do the cyan and violet. Notice that for tr(*W*_*z*_) the violet curve gives the exact optimum. (**c**) Overlap in the node ranking of the different driver node placement strategies. Color code is the same as in (**b**). The only highly significant overlap is between *w*_out_/*w*_in_ and tr(*W*_*z*_), while *λ*_min_(*W*_*z*_) and ${\rm{tr}}({W}_{z}^{-1})$ correspond to different node ranking patterns. Notice that none of the strategies orders nodes according to *w*_in_/*w*_out_ (mid panel). (**d**) Inverse correlation between the inertia at node *i*, *M*_*ii*_, and *w*_out_/*w*_in_ (correlation coefficient around −0.75 in average).

The system (7) has purely imaginary eigenvalues equal to ±*iω*_*j*_, *j* = 1, …, *n*, where *ω*_*j*_ are the natural frequencies of the oscillators. If ${{\rm{\Omega }}}^{2}={\rm{diag}}({\omega }_{1}^{2},\ldots ,{\omega }_{n}^{2})$ and Ψ = [*ψ*^1^ $\cdots$ *ψ*^*n*^] is the matrix of corresponding eigenvectors, then in the so-called modal basis the oscillators are decoupled and one gets the state space representation

8 $$\begin{array}{rcl}\dot{z} & = & {A}_{1}z+{B}_{1}u\\  & = & [\frac{0}{-{{\rm{\Omega }}}^{2}}|\frac{I}{0}]\,z+[\frac{0}{{{\rm{\Psi }}}^{T}\,{M}^{-1}\,B}]\,u\end{array}$$

where $z=[\frac{{{\rm{\Psi }}}^{-1}}{0}|\frac{0}{{{\rm{\Psi }}}^{-1}}]x$. See SI for the details. When a system has purely imaginary eigenvalues, the finite time reachability Gramian diverges as *t*_*f*_ → ∞. However, in the modal basis (8) the Gramian (here denoted *W*_*z*_) is diagonally dominant and linear in *t*_*f*_, hence as *t*_*f*_ grows it can be approximated by a diagonal matrix which can be computed explicitly^29^:

9 $${W}_{z}({t}_{f})\approx \sum _{j=1}^{n}\,\frac{{\beta }_{j}}{2{M}_{jj}^{2}}\,[\begin{array}{cccccc}\frac{{({\psi }_{j}^{1})}^{2}}{{\omega }_{1}^{2}} &  &  &  &  & \\  & \ddots  &  &  &  & \\  &  & \frac{{({\psi }_{j}^{n})}^{2}}{{\omega }_{n}^{2}} &  &  & \\  &  &  & {({\psi }_{j}^{1})}^{2} &  & \\  &  &  &  & \ddots  & \\  &  &  &  &  & {({\psi }_{j}^{n})}^{2}\end{array}]\,{t}_{f},$$

where *β*_*j*_ = 1 if the *j*-th input is present and 0 otherwise, and *M*_*jj*_ is the *j*-th diagonal entry of *M*, see SI for the details. For purely imaginary spectra (of which a network of coupled harmonic oscillators is a special case), using (9), the three measures of control energy adopted in this paper give rise to simple strategies for minimum energy driver nodes placement, which in some cases can be computed exactly for any *n* (for instance for the metric tr(*W*_*z*_), see SI). The three algorithms are explained in detail in the SI. Figure 6 shows that such strategies are always beating a random driver node placement, often by orders of magnitude.

Also *w*_out_/*w*_in_ is still a good heuristic for driver node placement strategy. This can be understood by observing that the model (6) is symmetric hence for it in- and out-degrees are identical. However, since *A*_*o*_ has rows rescaled by *M*^−1^, *w*_out_ is affected directly: when the inertia *M*_*ii*_ is big, the corresponding ${w}_{{\rm{out}}}(i)={\sum }_{j=1}^{n}\,{K}_{ji}/{M}_{ii}$ is small and viceversa. No specific effect is instead induced on *w*_in_. In the representation (7), selecting nodes according to *w*_out_/*w*_in_ means placing control inputs on the lighter masses, see Fig. 6(d). When the harmonic oscillators are decoupled (*L* = 0) then *m* < *n* means controllability is lost, but nevertheless selecting the nodes with least inertia as driver nodes maximizes the impact of a limited control authority (tr(*W*_*z*_) is maximized). A weak (and sparse) coupling allows to recover controllability, while the least inertia as optimal driver node strategy becomes suboptimal. When the coupling becomes stronger (for instance when the coupling graph is more connected) then the inertia of an oscillator is less significant as a criterion for selection of driver nodes: the modes of the system are now spread throughout the network and no longer localized on the individual nodes. As shown in Fig. S9, in a fully connected network of harmonic oscillators, driver node strategies based on *w*_out_/*w*_in_ and on tr(*W*_*z*_) tend to perform considerably worse for the other measures (*λ*_min_(*W*_*z*_) and ${\rm{tr}}({W}_{z}^{-1})$), while for a sparse graph (here ER graphs with *p* = 0.05), of the three explicit optimal driver node placement strategies available in this case, tr(*W*_*z*_) has a high overlap with *w*_out_/*w*_in_, see Fig. 6(b), while the other two tend to rank controls in somewhat different ways. Given that in this case we have three strategies that are (near) optimal for the chosen measure of control energy, the dissimilarity of the node rankings of these three strategies means that the driver node placement problem is heavily dependent on the way control energy is quantified.

### Application to minimum energy control of power grids

In the linear regime, power grids can be modeled as networks of weakly damped coupled harmonic oscillators^30^. The so-called swing equation corresponds in fact to the following network of damped and coupled harmonic oscillators

10 $$M\ddot{q}+D\dot{q}+Kq=Bu,$$

where *D* is the matrix of dampings which we assume to be proportional, that is, that in the modal basis *D*_1_ = Ψ^*T*^ *M*^−1^ *DM*^−1^ Ψ is diagonal. In the state space representation (7), one gets then

11 $$\dot{x}=[\frac{0}{-K\,{M}^{-1}}|\frac{I}{-D\,{M}^{-1}}]\,x+[\frac{0}{B}]\,u,$$

while in the modal basis

12 $$\dot{z}=[\frac{0}{-{{\rm{\Omega }}}^{2}}|\frac{I}{-{D}_{1}}]\,z+[\frac{0}{{{\rm{\Psi }}}^{T}\,{M}^{-1}\,B}]\,u.$$

**Figure 7:**
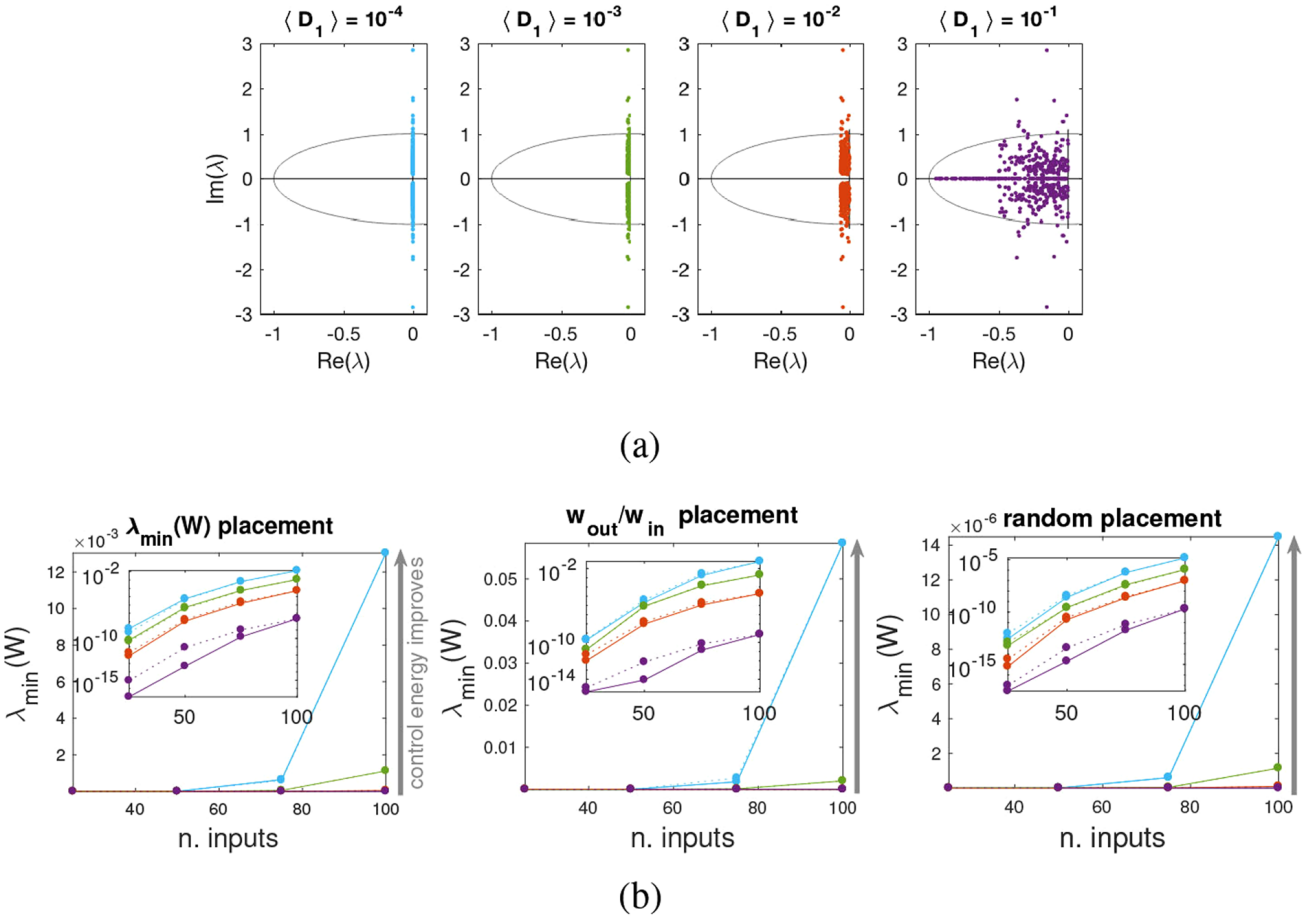
Minimum energy control of power grids for varying damping coefficients. (**a**) The eigenvalues of the state space system (11) for the North EU power grid^37^ with uniformly distributed masses (〈*M*_*ii*_〉 = 10) and damping coefficients that vary across 4 orders of magnitude. Since the system is always stable, *W*_*m*_ = *W*_*r*_. (**b**) Control energy for the metric *λ*_min_(*W*_*r*_) when the driver nodes are placed according to *λ*_min_(*W*_*r*_) (left panel), *w*_out_/*w*_in_ (mid panel), or randomly (right panel). The values of *λ*_min_(*W*_*r*_) corresponding to the 4 choices of damping made in (**a**) are shown in solid lines (same color code as in (**a**)), while in dotted lines the values of *λ*_min_(*W*_*z*_) are shown (suitably normalized to eliminate the explicit dependence from *t*_*f*_, see (9)). The insets show the same quantities in log scale. Values are all averages over 100 realizations. For all three driver node placement strategies, the performances worsen as the damping is increased. Comparing the three panels, *w*_out_/*w*_in_ performs similarly to *λ*_min_(*W*_*r*_), and both outperform a random placement by orders of magnitude.

The presence of damping makes the state update matrix stable, hence *W*_*m*_ = *W*_*r*_. For weak damping, the driver node selection strategies illustrated above can be applied to the model (11) and so can the method based on *w*_out_/*w*_in_. We have investigated the minimum energy control of several power grids listed in Table S2, varying the dampings across several orders of magnitude, see Fig. 7(a). As expected, for all of them the energy required to achieve controllability increases as the real part of the eigenvalues moves away from the imaginary axis, see Figs 7(b) and S10–S13. All strategies still beat a random driver node placement, even those based on the Gramian (9), formally valid only for undamped dynamics.

## Discussion

This paper contains two distinct contributions to the problem of understanding what determines the control energy required to completely control a complex network. The first is to show that the time constants of the free evolution of the system play a key role. Recall that in a linear unforced dynamical system the real part of the eigenvalues of *A* determines how fast/slow a system converges to the origin (stable eigenvalues, when real part of *λ*(*A*) is negative) or diverges to ∞ (unstable eigenvalues, when real part of *λ*(*A*) is positive). Such convergence/divergence speed grows with the absolute value of the real part of *λ*(*A*). In the complete controllability problem, both stable and unstable modes of *A* are gathered together, and all have to be “dominated” by the controls to achieve controllability. This result is valid regardless of the topology of the network, and of the number of controls used (provided, of course, that controllability is guaranteed). When the modes of the system are all slow, like when they are very close to the imaginary axis, then the energy needed to dominate them all is lower than when some of them are fast (i.e., the eigenvalues have large real part).

In the limit case of all eigenvalues of *A* being purely imaginary, we can also recover a known result from control theory affirming that controllability from any *x*_*o*_ to any *x*_*f*_ can be achieved in finite time by means of control inputs of bounded amplitude. As a matter of fact, an alternative approach used in control theory to take into account the control energy of a state transfer is to impose that the amplitude of the input stays bounded for all times (rather than the total energy), and to seek for conditions that guarantee controllability with such bounded controls^31–33^. Assume *u* ∈ Ω, with Ω a compact set containing the origin, for instance Ω = [−1, 1]^*m*^, where *m* is the number of control inputs. The constraint *u* ∈ Ω guarantees that we are using at all times a control which has an energy compatible with the physical constraints of our network. The consequence is, however, that reaching any point in ${{\mathbb{R}}}^{n}$ may require a longer time, or become unfeasible. In particular a necessary and sufficient condition for any point to be reachable from 0 in finite time when *u* ∈ Ω is that no eigenvalue of *A* has a negative real part, see SI. This is clearly connected with our previous considerations on the reachability problem without bounds on *u*: when all modes of *A* are unstable then the input energy required to reach any state from 0 is low (Fig. 1(a)) and becomes negligible for sufficiently long time horizons. On the contrary, transferring any state to 0 in finite time with *u* ∈ Ω is possible if and only if no eigenvalue of *A* has a positive real part. Also in this case the extra constraints on the input amplitude reflects the qualitative reasoning stated above and shown in Fig. 1(b). Also in the bounded control case, considering a generic transfer from any state *x*_*o*_ to any other state *x*_*f*_ means combining the two scenarios just described: formally a system is completely controllable from any *x*_*o*_ to any *x*_*f*_ in finite time and with bounded control amplitude *u* ∈ Ω if and only if all eigenvalues of *A* have zero real part, see SI for the details. The findings discussed above for *u* unbounded are completely coherent with this alternative approach to “practical controllability”.

The second contribution of this paper is a general strategy for driver node placement which reduces the control energy. Once the technical issues associated with minimal controllability can be neglected (in the sense that we are choosing sufficiently many inputs so as to guarantee controllability), a general criterion for controlling a network with a limited input energy is to drive the nodes having the maximal disembalance between their weighted outdegree and indegree. This driver node selection strategy has a direct topological interpretation, in terms of outdegree “dominance” (in a sense, it resembles the idea of dominating sets discussed in the literature^9^ although for directed edges only, as for undirected networks *r*_*w*_(*i*) = 1 ∀*i*). Our node ranking strategy has also another indirect topological interpretation. For random edge weights, nodes that have high *r*_*w*_ tend to be outdegree dominated, and hence to be associated to (topological) dilations, see Fig. 3(a). A set ${\mathscr{S}}$ of nodes forms a dilation if $|{\mathscr{S}}| > |T({\mathscr{S}})|$, where $T({\mathscr{S}})$ are the parent nodes of the nodes in ${\mathscr{S}}$ and $|{\mathscr{S}}|$ (and $|T({\mathscr{S}})|$) denotes set cardinality^1,34^. When seeking minimal (structural) controllability, dilations cannot appear, meaning that controls must be added to the children nodes in excess^34^. However, when minimal controllability is not the main concern and a number of driver nodes achieving controllability has already been selected, the strategy we are suggesting for reducing the control energy tends to favor nodes that are parent nodes of a dilation, rather than children. The rationale behind it is that in a dilation controlling parent nodes impacts several eigenmodes of the system (and hence reduces the control energy) while controlling children nodes enlarges the reachable subspace and for instance allows to discriminate among children of the same parents (and hence improves the structural controllability properties)^35^. We think that this trade-off could be responsible for the limited success in finding effective minimum control energy driver node strategies in the literature.

It has already been pointed out^11^ that guaranteeing controllability in a structural sense does not correspond to guaranteeing it in practice, as the required control energy can be exceedingly high. Our analysis of real-world networks confirms and strengthen this observation. Quite remarkably, we show that there are cases in which structural controllability fails, but nevertheless the control energy (which is theoretically infinite) turns out to be numerically lower that the one obtained in structurally controllable cases, even with equal numbers of controls. Such paradox follows from the fact that in both situations the Gramian is near-singular, which impacts measures like *λ*_min_(*W*_*m*_) and ${\rm{tr}}({W}_{m}^{-1})$. On the contrary, tr(*W*_*m*_) does not require *W*_*m*_ to be invertible, and in fact in nearly all networks we find that even with equal numbers of controls our *r*_*w*_-ranked driver node strategy outperforms the driver node selection based on structural controllability. This confirms the effectiveness of our node ranking strategy.

Obviously our analysis requires to assign sets of weights to the edges, not to rely exclusively on structural properties. The consistency in the results we find across all investigated real-world networks makes us confident that our observations are not spurious numerical artifacts. The conclusion one can draw from our quantitative analysis is that “binary” (yes/no) responses to the controllability question have limited value and should be taken with extreme care.

As can be seen comparing Fig. 5(a) (and Table 1) with Fig. S6(a), the topology drastically influences the minimal number of controls needed to achieve structural controllability. It also influences the minimum energy problem, and makes it difficult to disentangle the two aspects analytically. For instance, the transcriptional networks we consider (*E*. *coli*-*transcr* and *Yeast*-*transcr*) are essentially directed acyclic graphs, and as such require an extremely high number of driver nodes to achieve minimal controllability^1^. Nonetheless, by adding a few more driver nodes according to our *r*_*w*_ ranking, at least in *E*. *coli*-*transcr* the control energy can still be reduced by several orders of magnitude, meaning that even in these “extreme” topologies there is a lot to gain in terms of practical control by carefully selecting extra driver nodes. By comparing Figs S6 and 5(b), it is possible to realize that, rather than the indegree and outdegree distribution *per se*, it is the fraction of nodes having a high *r*_*w*_ (after the *m*_*c*_ nodes needed to guarantee controllability have been disregarded, violet curves in Fig. S6(b)) that determines the efficacy of our strategy. Although it is difficult to obtain a sharp classification, indicatively the networks that show a limited improvement with our driver node selection procedure (for instance *Yeast*-*transcr*, *US*-*airport* and *Wheat*-*trade*) correspond to those having a fairly limited number of nodes with high *r*_*w*_ in the *m*_*f*_ extra nodes being selected. Clearly a future step of our research is to extend our driver node strategy so as both minimal number of controls and minimum control energy are achieved at the same time.

Notice that our computation of weighted out/indegrees considers the total sum of weights in absolute value. When signs are taken into account in computing *w*_in_ and *w*_out_, then no significant improvement over random input placement is noticeable. This is connected to the quadratic nature of the Gramian.

Finally, it is worth emphasizing that for a dynamical system the concept of driver node is not intrinsic, but basis-dependent. In fact, just like the idea of adjacency matrix of a graph is not invariant to a change of basis in state space, so inputs associated to single nodes (i.e., to single state variables) in the original basis become scattered to all variables in another representation of the same system, see Fig. 6(a). Our calculations of driver nodes placement are always in the original basis, in which the adjacency matrix is expressed (for instance the representation (7) for the coupled harmonic oscillator, and (11) for the power grid). If we take a special basis in which the modes are decoupled (for instance the Jordan basis), then the contribution of the nodes to the modes (i.e., the eigenvectors of *A*) provide useful information for the investigation of minimum input energy problems. The topic is closely related to the so-called participation factors analysis in power networks^36^. Also quantities like *w*_in_ and *w*_out_ are basis-dependent and become nearly equal for instance if in (1) we pass to a Jordan basis. On the contrary, the eigenvalues of *A* are invariant to a change of basis. Hence as a general rule, the control energy considerations that are consequence of the time constants of the system (like the dependence on the real part of the eigenvalues illustrated in Fig. 2) are “more intrinsic” than those that follow from the particular basis representation we have available for a network.

## Electronic supplementary material

Supplementary Material
