# Supplementary material for: Minimum energy control for complex networks
Source: Sci Rep. 2018 Feb 16;8:3188. doi: 10.1038/s41598-018-21398-7 (PMC5816648; doi:10.1038/s41598-018-21398-7)
Supplement: Supplementary file 1 — Supplementary Material [file 41598_2018_21398_MOESM1_ESM.pdf]

SUPPLEMENTARY MATERIAL Minimum energy control for complex networks

Gustav Lindmark and Claudio Altafini*
Division of Automatic Control, Dept. of Electrical Engineering, Linköping University, SE-58183, Linköping, Sweden.

November 19, 2017

Contents

1 Methods **2**
1.1 Control energy: finite-time horizon formulation 2
1.2 Control energy: infinite-time horizon formulation 4
1.3 Mixed Gramian in infinite and finite time horizon 8
1.4 Controllability with bounded controls 10
1.5 Control of coupled harmonic oscillators 10

2 Datasets **16**

*Corresponding author: C. Altafini. Email: claudio.altafini@liu.se

1 Methods

1.1 Control energy: finite-time horizon formulation

Consider a linear system
$$\dot{x} = Ax + Bu \tag{S1}$$
where $x \in \mathbb{R}^n$ is the state vector, $A \in \mathbb{R}^{n\times n}$ is the state update matrix, $B \in \mathbb{R}^{n\times m}$ is the input matrix, and u is the m-dimensional input vector. The *reachable set* of (S1) in time t_f from x_o is the set

$$\mathcal{R}_{t_f}(x_o) = \{x \in \mathbb{R}^n \quad \text{s. .t.} \quad \exists\, u : [0,\, t_f] \to \Omega \;\; \text{s. t.} \;\; \phi(t_f, u, x_o) = x\}$$

where $\phi(t, u, x_o)$ is the solution of (S1) at time t with input u and Ω is the admissible set of control inputs, here $\Omega = \mathbb{R}^m$.

The system (S1) is *reachable* (or *controllable from the origin* [1]) in time t_f if any $x_f \in \mathbb{R}^n$ can be reached from 0 by some control $u \in \Omega$ in time t_f, i.e. if $\mathcal{R}_{t_f}(0) = \mathbb{R}^n$. It is said *controllable to the origin* if any $x_o \in \mathbb{R}^n$ can be brought to 0 by some control $u \in \Omega$ in time t_f. The system (S1) is said *completely controllable* in time t_f if $\mathcal{R}_{t_f}(x_o) = \mathbb{R}^n$ for any $x_o \in \mathbb{R}^n$.

Finite-time Gramians. The time-t_f reachability (or controllability from 0) Gramian is the symmetric matrix

$$W_r(t_f) = \int_0^{t_f} e^{A\tau} B B^T e^{A^T \tau} d\tau, \tag{S2}$$

while the time-t_f controllability to 0 Gramian (normally called the controllability Gramian, [1]) is

$$W_c(t_f) = \int_0^{t_f} e^{-A\tau} B B^T e^{-A^T \tau} d\tau. \tag{S3}$$

The two Gramians are positive definite whenever $(A,\, B)$ is controllable, and are related by
$$W_r(t_f) = e^{At_f} W_c(t_f) e^{A^T t_f}.$$
Similarly, for their inverses,

$$W_r^{-1}(t_f) = e^{-A^T t_f} W_c^{-1}(t_f) e^{-At_f}.$$

Finite-time control energy for state transfer. The transfer of the state from any x_o into any other x_f in time t_f can be accomplished by many controls. In order to quantify how costly a state transfer is on a system, one can choose to consider the control input that minimizes the input energy, i.e., the functional

$$\mathcal{E}(t_f) = \int_0^{t_f} \|u(\tau)\|^2 d\tau. \tag{S4}$$

Such control can be computed explicitly [1] as

$$u(t) = B^T e^{A^T(t_f-t)} W_r^{-1}(t_f)(x_f - E^{At_f} x_o), \qquad t \in [0,\, t_f] \tag{S5}$$

and the corresponding transfer cost as

$$\mathcal{E}(t_f) = (x_f - e^{At_f} x_o)^T W_r^{-1}(t_f)(x_f - e^{At_f} x_o). \tag{S6}$$

To be more precise on how the control energy is formed, we have to split the state transfer energy (S6) into subtasks:

1. $x_o = 0$ (reachability problem)

$$\Longrightarrow \quad \mathcal{E}_r(t_f) = x_f^T W_r^{-1}(t_f) x_f;$$

2. $x_f = 0$ (controllability to 0 problem)

$$\Longrightarrow \quad \mathcal{E}_c(t_f) = x_o^T e^{A^T t_f} W_r^{-1}(t_f) e^{At_f} x_o = x_o^T W_c^{-1}(t_f) x_o.$$

In particular, both $W_r(t_f)$ and $W_c(t_f)$ enter into the cost function. Notice that, to quantify the amount of control energy of these problems we need to compute the inverse of $W_r(t_f)$ and $W_c(t_f)$.

Let us look at how the stability/instability of the eigenvalues influences the two costs $\mathcal{E}_r(t_f)$ and $\mathcal{E}_c(t_f)$.

- If A is stable (i.e., $\mathrm{Re}[\lambda(A)] < 0$), then "escaping from 0" (i.e., the reachability problem) requires more energy than transferring to 0, (i.e., the controllability to 0 problem) because the modes of A naturally tend to converge to 0.

- If A is antistable (i.e., $\mathrm{Re}[\lambda(A)] > 0$, $-A$ is stable), then the opposite considerations are valid: the modes of A tend to amplify the magnitude of the state, simplifying the reachability problem but complicating the controllability to 0 problem.

- If A has eigenvalues with both negative and positive real part, the two situations coexist.

Hence computing a plausible measure of control energy for a generic state transfer $x_o \to x_f$ requires to take into account the "difficult" directions of both cases.

Gramian-based measures of control energy. Expression like $\mathcal{E}(t_f)$ depend on the choice of x_o and x_f. In control theory, it has been known for a long time that it is possible to obtain more objective estimates of the control energy by computing figures of merits based on the Gramian [18]. The following metrics are often used:

1. $\lambda_{\min}(W_r) = \frac{1}{\lambda_{\max}(W_r^{-1})}$: the min eigenvalue of the Gramian is a worst-case metric, equal to the inverse of the max eigenvalue of W_r^{-1}. $\lambda_{\max}(W_r^{-1})$ estimates the energy required to move along the direction which is most difficult to control, hence when $\lambda_{\max}(W_r^{-1})$ descreases the control energy decreases. In turn, the control energy decreases when $\lambda_{\min}(W_r)$ increases.

2. $\text{tr}(W_r)$: the trace of the Gramian is inversely proportional to the average energy required to control a system, hence when $\text{tr}(W_r)$ increases the control energy decreases.

3. $\text{tr}(W_r^{-1})$: the trace of the inverse of the Gramian is proportional to the average energy needed to control the system, i.e., the control energy decreases with $\text{tr}(W_r^{-1})$.

In summary, minimizing the control energy means maximizing the first and second measure or minimizing the third.

1.2 Control energy: infinite-time horizon formulation

When $t_f \to \infty$, then $\mathcal{E}(t_f)$ converges (or diverges) to a quantity

$$\mathcal{E} = \int_0^\infty \|u(\tau)\|^2 d\tau, \tag{S7}$$

and so do $\mathcal{E}_r(t_f)$ and $\mathcal{E}_c(t_f)$.

When $t_f \to \infty$, both Gramians become infinite-time integrals, which may be convergent or divergent, depending on the modes of A. If A stable, then

$$W_r = \int_0^\infty e^{A\tau} B B^T e^{A^T \tau} d\tau \tag{S8}$$

exists finite and it is positive definite if (A, B) controllable. If instead A is antistable, then it is

$$W_c = \int_0^\infty e^{-A\tau} B B^T e^{-A^T \tau} d\tau \tag{S9}$$

to exist finite and positive definite when (A, B) controllable. In the mixed eigenvalues cases the two expression (S8) and (S9) both diverge, although their inverses both exist (see below): W_r^{-1} depends only on the stable modes, and W_c^{-1} only on the unstable modes.

In an expression like (S7), for $t_f \to \infty$ the "cross-terms" $x_o^T e^{At_f} W_r^{-1}(t_f) x_f$ vanish when A is stable (because $e^{At_f} \to 0$ when $t_f \to \infty$). They also vanish when A is antistable because the same cross terms can also be written as $x_o^T W_c^{-1}(t) e^{-At_f} x_f$, using the Controllability Gramian $W_c(t_f)$. In fact now $e^{-At_f} \to 0$ when $t_f \to \infty$.

Controllability to 0 in the infinite-time horizon. Let us observe what happens for instance to the controllability to 0 problem according to the eigenvalues of A.

- If A is stable, then in correspondence of $u = 0$, $\lim_{t\to\infty} x(t) = 0$ for all x_o, meaning that the controllability to 0 problem can be solved with zero energy $\mathcal{E}_c = 0$. Furthermore, the integral (S2) converges to (S8), whose value can be computed solving the following Lyapunov equation:

  $$A W_r + W_r A^T + B B^T = 0. \tag{S10}$$

  Such a solution always exists and it is $W_r > 0$ (positive definite) if the pair (A, B) is controllable.

- When instead A is antistable, then the integral (S2) diverges as $t_f \to \infty$. Hence the solution with $u = 0$ is no longer feasible as all modes are unstable (and diverge as soon as $x_o \neq 0$), meaning that to find a minimizer of (S7) we have to proceed in some other way. Since (A, B)

controllable, we can determine $u(t)$ as if we were computing a stabilizing feedback law, i.e., expressing $u(t)$ as a function of the state $x(t)$ so that the resulting closed loop system converges to 0 asymptotically. Such a feedback law can be computed solving in P an algebraic Riccati equation (ARE)

$$P(-A) + (-A^T)P + PBB^TP = 0. \tag{S11}$$

Such ARE admits a positive definite solution P, which can in turn be computed solving in L the Lyapunov equation (in $-A$, which is stable, hence a solution $L > 0$ always exists)

$$(-A)L + L(-A^T) + BB^T = 0, \tag{S12}$$

and then setting $P = L^{-1}$. It can be verified directly that the controllability Gramian W_c in (S9) is one such solution L, i.e., $L = W_c$ solves (S12). Correspondingly we obtain $P = W_c^{-1}$. From the theory of linear-quadratic regulators (in particular [24], Ch. 10) the feedback controller

$$u = -B^TPx(t) \tag{S13}$$

guarantees stability of the closed-loop system

$$\dot{x} = (A - BB^TP)x$$

i.e., $A - BB^TP$ is a stable matrix. The feedback law (S13) also minimizes the input energy (S7) which is equal to $\mathcal{E}_c = x_o^TW_c^{-1}x_o$.

- When A has eigenvalues with both positive and negative real part and no purely imaginary eigenvalues, then the two situations described above occur simultaneously. Assume A is split into two diagonal blocks, one consisting of only eigenvalues with negative real part and the second only of eigenvalues of positive real part. This can always be achieved through a change of basis [27]. Split B and $x(t)$ accordingly:

$$x = \begin{bmatrix} x_1 \\ x_2 \end{bmatrix}, \quad A = \begin{bmatrix} A_1 & 0 \\ 0 & A_2 \end{bmatrix}, \quad B = \begin{bmatrix} B_1 \\ B_2 \end{bmatrix}, \qquad \begin{array}{l} \mathrm{Re}[\lambda(A_1)] < 0 \\ \mathrm{Re}[\lambda(A_2)] > 0 \end{array} \tag{S14}$$

In the infinite time horizon, the $u = 0$ control steers optimally the x_1 subvector, while for the x_2 part a feedback controller provides the

energy-minimizing solution. From (S11) we obtain that the ARE has solution

$$P = \begin{bmatrix} 0 & 0 \\ 0 & P_2 \end{bmatrix}$$

where P_2 solves the ARE for the $(A_2,\, B_2)$ subsystem. Hence the control input

$$u = -BB^T P = -BB^T \begin{bmatrix} 0 & 0 \\ 0 & W_{2,c}^{-1} \end{bmatrix}$$

achieves a transfer to the origin with minimal energy cost equal to $\mathcal{E}_c = x_o^T P x_o = x_{2,o}^T W_{2,c}^{-1} x_{2,o}$. Furthermore, combining (S10) and (S12), we have that for (S14) the following two Lyapunov equations must hold simultaneously:

$$A_1 W_{1,r} + W_{1,r} A_1^T + B_1 B_1^T = 0 \tag{S15a}$$

$$(-A_2) W_{2,c} + W_{2,c}(-A_2^T) + B_2 B_2^T = 0 \tag{S15b}$$

Reachability in the infinite-time horizon. Let us now consider the reachability problem (i.e., controllability from 0). Now the roles of stable and unstable eigenvalues are exchanged.

- If A stable, reachability requires an active control (here a destabilizing state feedback law) in order to steer $x(t)$ out of the origin. The energy-optimal solution consists in choosing $u = -B^T P x(t)$ with $P > 0$ solution of the ARE

  $$PA + A^T P + PBB^T P = 0 \tag{S16}$$

  or, equivalently, $P = K^{-1}$ with K solution of the Lyapunov equation

  $$AK + KA^T + BB^T = 0. \tag{S17}$$

  A is stable, hence $K > 0$ solving (S17) and $P > 0$ solving (S16) always exist. The resulting closed loop matrix $A - BB^T P$ must be antistable. From (S10) and (S17) it can also be $K = W_r$, the reachability Gramian.

- If A antistable, then $u = 0$ is the minimal energy controller (an infinitesimal amount energy at $t = 0$ is enough to "kick" the system towards the right direction x_f when initialized in $x_o = 0$; this amount of energy is negligible in the infinite time horizon considered here). Since $-A$ stable, a Lyapunov equation like (S12) holds, with solution $L = W_c$.

- When A has eigenvalues with both positive and negative real part and no purely imaginary eigenvalues, then a decomposition like (S14) can be obtained through a change of basis. The complete ARE has now solution

$$P = \begin{bmatrix} P_1 & 0 \\ 0 & 0 \end{bmatrix} = \begin{bmatrix} W_{1,r}^{-1} & 0 \\ 0 & 0 \end{bmatrix},$$

and the controller achieving the transfer with minimal energy is

$$u = -BB^T \begin{bmatrix} W_{1,r}^{-1} & 0 \\ 0 & 0 \end{bmatrix},$$

for an amount of energy equal to

$$\mathcal{E}_r = x_{1,f}^T W_{1,r}^{-1} x_{1,f}$$

The decomposition (S14) also in this case induce a pair of Lyapunov equations identical to (S15).

1.3 Mixed Gramian in infinite and finite time horizon

In order to assemble the considerations of the previous sections, it is useful to introduce a third Gramian which we call *mixed Gramian*, W_m and which gathers the directions difficult to control of both the reachability and the controllability to 0 problems. This mixed Gramian is used extensively in the paper.

Infinite-time horizon mixed Gramian Assume that the spectrum of A contains k, $0 \leqslant k \leqslant n$, eigenvalues with negative real part, and $n-k$ eigenvalues with positive real part (and no purely imaginary eigenvalues). Then, as already mentioned above, there exist a change of basis V bringing A into the form (S14):

$$\begin{bmatrix} \bar{A}_1 & 0 \\ 0 & \bar{A}_2 \end{bmatrix} = VAV^{-1} \tag{S18}$$

and, correspondingly,

$$\begin{bmatrix} \bar{B}_1 \\ \bar{B}_2 \end{bmatrix} = VB$$

with $\mathrm{Re}[\lambda(\bar{A}_1)] < 0$ and $\mathrm{Re}[\lambda(\bar{A}_2)] > 0$. In the new basis, the two Lyapunov equations (S15) hold, which can be rewritten as

$$\begin{bmatrix} \bar{A}_1 & 0 \\ 0 & -\bar{A}_2 \end{bmatrix} \bar{W} + \bar{W} \begin{bmatrix} \bar{A}_1 & 0 \\ 0 & -\bar{A}_2 \end{bmatrix}^T + \begin{bmatrix} \bar{B}_1 \bar{B}_1^T & 0 \\ 0 & \bar{B}_2 \bar{B}_2^T \end{bmatrix} = 0 \tag{S19}$$

with

$$\bar{W}_m = \begin{bmatrix} \bar{W}_{1,r} & 0 \\ 0 & \bar{W}_{2,c} \end{bmatrix}$$

the mixed Gramian. Following [27], the expression of the mixed Gramian in the original basis is $W_m = V^{-1} \bar{W}_m V^{-T}$. By construction, the mixed Gramian matrix W_m always exists when A has no purely imaginary eigenvalues, and it summarizes the infinite-horizon contribution of the stable eigenvalues to the reachability problem and of the unstable eigenvalues to the controllability to 0 problem (i.e., all cases leading to a high control energy).

Control energy measures for the mixed Gramian The three control energy metrics introduced above can be computed also for the mixed Gramian, with an analogous interpretation. In fact $\lambda_{\min}(W_m)$, $\mathrm{tr}(W_m)$ and $\mathrm{tr}(W_m^{-1})$ are the three measures that are mostly used in the paper to quantify the control energy.

Finite-time horizon mixed Gramian Using the insight given by the previous arguments, it is possible to construct also a finite-time mixed Gramian, which weights only the modes that are difficult to control in the two state transfer problems. In the basis in which A is split into stable and antistable diagonal blocks, (S18), this is given by

$$\bar{W}_m(t_f) = \begin{bmatrix} \bar{W}_{1,r}(t_f) & 0 \\ 0 & \bar{W}_{2,c}(t_f) \end{bmatrix}$$

where $\bar{W}_{1,r}(t_f)$ and $\bar{W}_{2,c}(t_f)$ are the equivalent of (S2) and (S3) for the two subsystems $(\bar{A}_1, \bar{B}_1)$ and $(\bar{A}_2, \bar{B}_2)$. An equation like (S19) has no coupling terms between the two subsystems (i.e., terms of the form $\bar{B}_1 \bar{B}_2$). These terms disappear asymptotically, but transiently they give a contribution, hence the finite-time formulation of $\bar{W}_m(t_f)$ is only an approximation. In the original basis, $W_m(t_f) = V^{-1} \bar{W}_m(t_f) V^{-T}$, and the input energy is

$$\mathcal{E}_m(t_f) = \begin{bmatrix} x_{1,f}^T & x_{2,o}^T \end{bmatrix} W_m(t_f)^{-1} \begin{bmatrix} x_{1,f} \\ x_{2,o} \end{bmatrix}.$$

Clearly a proxy for this quantity is obtained by simply flipping the sign the real part of the unstable eigenvalues of A and considering only the reachability problem on the resulting stable system. Equivalently, all eigenvalues can be made unstable, and the controllability to 0 problem considered. More details and explicit formulas for the finite-time horizon are provided in e.g. [14, 22].

1.4 Controllability with bounded controls

Consider the system (S1). Assume $u \in \Omega$, where Ω is a compact set of $\mathbb{R}^m$ containing the origin in its interior. Assume $(A,\ B)$ is controllable. Then we have the following, see [13, 5] and [23], p. 122.

- A necessary and sufficient condition for the origin to be steered to any point of $\mathbb{R}^n$ in finite time (i.e., the reachability problem) is that no eigenvalue of A has negative real part.
- A necessary and sufficient condition for any point of $\mathbb{R}^n$ to be steered to the origin in finite time (i.e., the controllability to 0 problem) is that no eigenvalue of A has positive real part.

Combining the two:

- A necessary and sufficient condition for complete controllability (from any point x_o to any point x_f) in finite time is that all eigenvalues have zero real part.

1.5 Control of coupled harmonic oscillators

A network of n coupled harmonic oscillators can be written as a system of second order differential equations

$$M_{ii}\ddot{q}_i + (k_i + \sum_{j=1}^{n} k_{ij})q_i - \sum_{j=1}^{n} k_{ij}q_j = \beta_i u_i, \qquad i = 1, \ldots, n, \tag{S20}$$

where $M_{ii} > 0$ is the mass of the i-th oscillator, $k_i \geqslant 0$ its stiffness, $k_{ij} \geqslant 0$ the coupling stiffness between the i-th and j-th oscillators, and $\beta_i \in \{0,\ 1\}$ indicates the presence or absence of a forcing term in the i-th oscillator. In matrix form, (S20) can be rewritten as

$$M\ddot{q} + Kq = Bu, \tag{S21}$$

where $M = M^T = \text{diag}(M_{ii}) > 0$ is the mass matrix, $K = K^T \geqslant 0$ the stiffness matrix, and B is a $n \times m$ matrix whose columns are the elementary vectors corresponding to the $\beta_i = 1$. When $u = 0$, the solutions of (S21) have the form $q = \phi e^{i\omega t}$ in correspondence of the pairs ω_j and ϕ^j, $j = 1, \ldots, n$, that are the solutions of the generalized eigenvalues/eigenvector equation

$$(-\omega^2 M + K)\phi = 0. \tag{S22}$$

The ω_j are called the natural frequencies of (S21). Denote

$$\Phi = \begin{bmatrix} \phi^1 & \ldots & \phi^n \end{bmatrix}$$

the matrix of eigenvectors. Φ can be used to pass to a so-called modal basis, in which the oscillators are decoupled. In fact, it can be verified directly that in correspondence of the change of basis $q_1 = \Phi^{-1} q$, $M_1 = \Phi^T M \Phi$ and $K_1 = \Phi^T K \Phi$ are both diagonal matrices, hence

$$M_1 \ddot{q}_1 + K_1 q_1 = \Phi^T B u$$

has decoupled dynamics (but coupled inputs).

The state space representation of (S21) is $2n$ dimensional. If

$$x = \begin{bmatrix} M & 0 \\ 0 & M \end{bmatrix} \begin{bmatrix} q \\ \dot{q} \end{bmatrix}, \tag{S23}$$

then

$$\dot{x} = A_o x + B_o u = \left[\begin{array}{c|c} 0 & I \\ \hline -KM^{-1} & 0 \end{array}\right] x + \left[\begin{array}{c} 0 \\ \hline B \end{array}\right] u. \tag{S24}$$

In terms of the state space model (S24), the eigenvalues are $\lambda_j = \pm i\omega_j$, $j = 1, \ldots, n$, of eigenvectors

$$v_j = \left[\begin{array}{c} \psi^j \\ \hline \omega_j \psi^j \end{array}\right]$$

where $\psi^j = M\phi^j$, from which the purely oscillatory nature of A_o is evident. If we denote

$$\Omega^2 = \begin{bmatrix} \omega_1^2 & & & \\ & \omega_2^2 & & \\ & & \ddots & \\ & & & \omega_n^2 \end{bmatrix},$$

then, from (S22), $M^{-1}K\Phi = \Phi\Omega$ which implies

$$\begin{aligned}\Omega^2 &= \Phi^{-1}M^{-1}K\Phi \\ &= \Phi^{-1}M^{-1}\Phi^{-T}\Phi^{T}K\Phi \\ &= M_1^{-1}K_1.\end{aligned}$$

If $\Psi = M\Phi$, the state space representation in the modal basis

$$z = Tx = \left[\begin{array}{c|c} \Psi^{-1} & 0 \\ \hline 0 & \Psi^{-1} \end{array}\right] x$$

is given by

$$\dot{z} = A_1 z + B_1 u = \left[\begin{array}{c|c} 0 & I \\ \hline -\Omega^2 & 0 \end{array}\right] z + \left[\begin{array}{c} 0 \\ \hline \Psi^{-1}B \end{array}\right] u. \tag{S25}$$

From $M_1 = \Psi^T M^{-1} \Psi$, one gets $\Psi^{-1}B = M_1^{-1}\Psi^T M^{-1} B$.

A key advantage of the modal representation is that the reachability Gramian of the pair (A_1, B_1) can be computed explicitly. As a matter of fact, when the eigenvalues are on the imaginary axis, there is no need to resort to the mixed Gramian introduced earlier. As the integral (S2) (or (S3)) diverges, the infinite-time Gramian cannot be computed. However, in the modal basis z, $W_z(t_f)$ (or more precisely $W_{z,r}(t_f)$) is diagonally dominant, and for t_f sufficiently long it can be approximated by its diagonal terms. These terms are computed explicitly in [2]:

$$(W_z(t_f))_{jj} = \begin{cases} \frac{\left(M_1^{-1}\Psi^T M^{-1} B B^T M^{-1} \Psi M_1^{-T}\right)_{jj} t_f}{2\omega_j} & \text{for } 1 \leqslant j \leqslant n \\ \frac{\left(M_1^{-1}\Psi^T M^{-1} B B^T M^{-1} \Psi M_1^{-T}\right)_{jj} t_f}{2} & \text{for } n+1 \leqslant j \leqslant 2n. \end{cases}$$

If we assume that the mass matrix M is diagonal, then it is always possible to choose Ψ so that $M_1 = I$ and K_1 diagonal, by suitably rescaling the eigenvectors ψ^j. In this case

$$B_1 B_1^T = \left[\begin{array}{c} 0 \\ \hline \Psi^T M^{-1} B \end{array}\right] \left[\begin{array}{c|c} 0 & B^T M^{-1}\Psi \end{array}\right] = \left[\begin{array}{c|c} 0 & 0 \\ \hline 0 & \Psi^T M^{-1} B B^T M^{-1} \Psi \end{array}\right],$$

and the Gramian is determined by the lower diagonal block. When the columns of B are elementary vectors as in our case, the product $\Psi^T M^{-1} B B^T M^{-1} \Psi$

can be written explicitly as sum of rank-1 matrices:

$$\Psi^T M^{-1} B B^T M^{-1} \Psi = \sum_{j=1}^{n} \frac{\beta_j}{M_{jj}^2} \begin{bmatrix} \psi_j^1 \\ \vdots \\ \psi_j^n \end{bmatrix} \begin{bmatrix} \psi_j^1 & \dots & \psi_j^n \end{bmatrix}$$

(only m of the n factors $\beta_j \in \{0, 1\}$ are nonzero) and its diagonal entries are

$$\text{diag}(\Psi^T M^{-1} B B^T M^{-1} \Psi) = \sum_{j=1}^{n} \frac{\beta_j}{M_{jj}^2} \begin{bmatrix} (\psi_j^1)^2 & & \\ & \ddots & \\ & & (\psi_j^n)^2 \end{bmatrix}. \qquad \text{(S26)}$$

Hence the expression for the Gramian in the modal basis is

$$W_z(t_f) \approx \begin{bmatrix} \sum_{j=1}^{n} \frac{\beta_j}{M_{jj}^2} \frac{(\psi_j^1)^2}{2\omega_1^2} & & & & & \\ & \ddots & & & & \\ & & \sum_{j=1}^{n} \frac{\beta_j}{M_{jj}^2} \frac{(\psi_j^n)^2}{2\omega_n^2} & & & \\ & & & \sum_{j=1}^{n} \frac{\beta_j}{M_{jj}^2} \frac{(\psi_j^1)^2}{2} & & \\ & & & & \ddots & \\ & & & & & \sum_{j=1}^{n} \frac{\beta_j}{M_{jj}^2} \frac{(\psi_j^n)^2}{2} \end{bmatrix} t_f.$$

Notice the linearity in t_f, meaning that all components diverge to ∞ with the same speed when $t_f \to \infty$. This expression can be used to compute the various measures of control energy we have adopted in the paper, and hence to optimize the driver node placement problem. For instance, selecting inputs according to $\lambda_{\min}(W_z)$ amounts to solving the following MILP max-min problem:

$$\begin{aligned} \max_{\beta_j} \min_{i} \sum_{j=1}^{n} \frac{\beta_j}{M_{jj}^2} (\psi_j^i)^2 & \\ \text{subject to } \sum_{j=1}^{n} \beta_j &= m \\ \beta_j &\in \{0, 1\} \end{aligned}$$

which can be solved exactly only for systems of moderate size. However, efficient heuristics can be derived for it, such as Algorithm 1.

Algorithm 1 Driver node placement that maximizes $\lambda_{\min}(W_z)$.

Input:

$$y^i = \frac{1}{M_{ii}^2}\begin{bmatrix} \frac{(\psi_i^1)^2}{2\omega_1^2} & \dots & \frac{(\psi_i^n)^2}{2\omega_n^2} & \frac{(\psi_i^1)^2}{2} & \dots & \frac{(\psi_i^n)^2}{2} \end{bmatrix}, \quad i = 1, \dots, n$$

1. Choose $\hat{i} = \text{argmax}_i(-\|y^i\|_\infty) = \text{argmax}_i(\min_k y_k^i)$
   - $y^s = y^{\hat{i}}$
   - $\mathcal{I} = \{1, 2, \dots, n\} \setminus \{\hat{i}\}$
   - $\mathcal{O} = \{\hat{i}\}$
2. For $c = 2, 3, \dots, m$
   - compute $\hat{j} = \text{argmax}_{j\in\mathcal{I}}(-\|y^s + y^j\|_\infty)$
   - $y^s = y^s + y^{\hat{j}}$
   - $\mathcal{I} = \mathcal{I} \setminus \{\hat{j}\}$
   - $\mathcal{O} = \mathcal{O} \cup \{\hat{j}\}$

Output: $\mathcal{O}$

If instead we choose to maximize the $\text{tr}(W_z)$, then we get

$$\begin{aligned} \max_{\beta_j} \sum_{i=1}^n \sum_{j=1}^n \frac{\beta_j}{M_{jj}^2} \frac{(\psi_j^i)^2(1+\omega_i^2)}{2\omega_i^2} \\ \text{subject to } \sum_{j=1}^n \beta_j = m \\ \beta_j \in \{0, 1\} \end{aligned}$$

Since $\text{tr}(W_z)$ is linear in the β_j, this is a linear optimization problem, hence solvable exactly and efficiently for any n. Finally, also for the minimization

of $\mathrm{tr}(W_z^{-1})$

$$\min_{\beta_j} \mathrm{tr}(W_z^{-1})$$
$$\text{subject to } \sum_{j=1}^{n} \beta_j = m$$
$$\beta_j \in \{0,\, 1\}$$

an efficient heuristic can be set, as outlined in Algorithm 2.

Algorithm 2 Driver node placement that minimizes $\mathrm{tr}(W_z^{-1})$.

Input:

$$y^i = \frac{1}{M_{ii}^2} \begin{bmatrix} \frac{(\psi_i^1)^2(1+\omega_1^2)}{2\omega_1^2} & \dots & \frac{(\psi_i^n)^2(1+\omega_n^2)}{2\omega_n^2} \end{bmatrix}, \quad i = 1, \dots, n$$

1. Choose $\hat{i} = \mathrm{argmax}_i \sum_{k=1}^{n} \frac{1}{y_k^i}$
   - $y^s = y^{\hat{i}}$
   - $\mathcal{I} = \{1,\, 2, \dots, n\} \setminus \{\, \hat{i}\,\}$
   - $\mathcal{O} = \{\, \hat{i}\,\}$
2. For $c = 2,\, 3, \dots, m$
   - compute $\hat{j} = \mathrm{argmax}_{j \in \mathcal{I}} \sum_{k=1}^{n} \frac{1}{y_k^s + y_k^j}$
   - $y^s = y^s + y^{\hat{j}}$
   - $\mathcal{I} = \mathcal{I} \setminus \{\, \hat{j}\,\}$
   - $\mathcal{O} = \mathcal{O} \cup \{\, \hat{j}\,\}$

Output: $\mathcal{O}$

Looking at an expression like (S26), it is possible to understand what kind of behavior yields good controllability properties to certain driver nodes. For instance when measuring according to $\lambda_{\min}(W_z)$, from (S26), the columns of Ψ^T express how nodes in the original basis are spread among the state

variables in the modal basis. What is needed is an eigenbasis such that the j-th component of all eigenvectors has "support" on all the directions of the state space, i.e., all $\psi_j^1, \ldots, \psi_j^n$ are nonvanishing and possibly all as large as possible. Since W_z is approximated well by a diagonal matrix, the "coverage" effect of control inputs is additive, hence choosing a pair of controls i and j for which the sum of $\{\psi_i^k\}_{k=1\ldots,n}$ and $\{\psi_j^k\}_{k=1\ldots,n}$ has all components that are as large as possible guarantees an improvement in the control cost with respect to taking only one of the two inputs.

Notice that although M and K are both symmetric, KM^{-1} need not be, hence Ψ need not be an orthogonal matrix. It can be rendered orthogonal if a slightly different modal basis is chosen, see [10] for the details. In that case $\Psi^T = \Psi^{-1}$ i.e., the "coverage" discussed here is given by the left eigenvectors of (S22), condition sometimes considered in the literature [11].

Another basis for the state space that can be used in place of (S23) is given by $\tilde{x} = \begin{bmatrix} q \\ \dot{q} \end{bmatrix}$. With this choice, the state space realization is

$$\dot{\tilde{x}} = \tilde{A}_o \tilde{x} + \tilde{B}_o u = \left[\begin{array}{c|c} 0 & I \\ \hline -M^{-1}K & 0 \end{array}\right] \tilde{x} + \left[\begin{array}{c} 0 \\ \hline M^{-1}B \end{array}\right] u. \tag{S27}$$

It is straightforward to verify that in this basis the roles of w_{in} and w_{out} are exchanged, hence a criterion for driver node selection becomes ranking according to $w_{\text{in}}/w_{\text{out}}$ (instead of $w_{\text{out}}/w_{\text{in}}$).

2 Datasets

The majority of the networks listed in Table 1 of the paper is composed of a large connected component, plus a number of other very small connected components. In our study, only the largest connected component is considered. The following networks are considered:

- Biology, transcriptional networks
  - *E.coli-transcr.*: Gene regulatory network of E.coli, downloaded from RegulonDB database (http://regulondb.ccg.unam.mx). See [9].
  - *Yeast-transcr.*: Gene regulatory network of the yeast S.cerevisiae originally developed in [17].

- Biology, metabolic networks
  - *E.coli-metab*: Metabolic network of E.coli, from [21].
  - *Yeast-metab*: Metabolic network of S.cerevisiae. Assembled from the list of reactions in [8].
- Biology, signaling networks
  - *Macrophage*: The molecular interaction map of a macrophage obtained by [19].
  - *Toll-like*: Signaling network for the Toll-like receptor. Assembled from [20].
- Eoclogical, Food-webs
  - *Foodweb-Florida*: Trophic dynamics in a south Florida ecosystem, from Pajek collection, see also [25].
  - *Foodweb-mangdry*: Mangrove Estuary, Dry Season, from Pajek collection [3].
- Social
  - *Moreno Highschool*: This directed network contains friendships between boys in a small highschool in Illinois in 1957/58. Edges represent friendship. From [6].
  - *Advogato*: Advogato is an online community platform for developers of free software launched in 1999. Nodes are users of Advogato and the directed edges represent trust relationships, see [15].
- Transport and Trade
  - *US Airport*: This is the directed network of flights between US airports in 2010. Each edge represents a connection from one airport to another. Compiled from the blog post "Why Anchorage is not (that) important: Binary ties and Sample selection", and downloadable from https://toreopsahl.com/datasets/#usairports.
  - *Wheat-trade*: Network representing trade relationships between countries from the Food and Agricultural Organization of the United Nations, see [7].

The power networks used in the second part of the paper are listed in Table S1. All nodes are treated equally, regardless of their function as generators or loads in the real grid.

Network	nodes	edges	source
North EU power grid	236	320	[16]
IEEE 300 test grid	300	409	https://www.ee.washington.edu/research/pstca/
French power grid	1888	2308	[12]
USA power grid	4941	6591	[26]

Table S1: Power grids used in this study.

References

[1] P.J. Antsaklis and A.N. Michel. *Linear Systems.* Birkhäuser Boston, 2005.

[2] Ami Arbel. Controllability measures and actuator placement in oscillatory systems. *International Journal of Control*, 33(3):565–574, 1981.

[3] V. Batagelj and A. Mrvar. Pajek data sets. http://pajek.imfm.si/doku.php?id=data:index. Accessed: 2016-09-21.

[4] Béla Bollobás, Christian Borgs, Jennifer Chayes, and Oliver Riordan. Directed scale-free graphs. In *Proceedings of the Fourteenth Annual ACM-SIAM Symposium on Discrete Algorithms*, SODA '03, pages 132–139, Philadelphia, PA, USA, 2003. Society for Industrial and Applied Mathematics.

[5] R. F. Brammer. Controllability in linear autonomous systems with positive controllers. *SIAM J of Control*, 10:339–353, 1972.

[6] J.S. Coleman. *Introduction to mathematical sociology.* Free Press of Glencoe, 1964.

[7] M. De Domenico, V. Nicosia, A. Arenas, and V. Latora. Structural reducibility of multilayer networks. *Nat Commun*, 6, 2015.

[8] Jochen Förster, Iman Famili, Patrick Fu, Bernhard Ø. Palsson, and Jens Nielsen. Genome-scale reconstruction of the *Saccharomyces cerevisiae* metabolic network. *Genome Res.*, 13(2):244–253, 2003.

[9] Socorro Gama-Castro, Heladia Salgado, Martin Peralta-Gil, Alberto Santos-Zavaleta, Luis Muiz-Rascado, Hilda Solano-Lira, Vernica Jimenez-Jacinto, Verena Weiss, Jair S. Garca-Sotelo, Alejandra Lpez-Fuentes, Liliana Porrn-Sotelo, Shirley Alquicira-Hernndez, Alejandra Medina-Rivera, Irma Martnez-Flores, Kevin Alquicira-Hernndez, Ruth Martnez-Adame, Csar Bonavides-Martnez, Juan Miranda-Ros, Araceli M. Huerta, Alfredo Mendoza-Vargas, Leonardo Collado-Torres, Blanca Taboada, Leticia Vega-Alvarado, Maricela Olvera, Leticia Olvera, Ricardo Grande, Enrique Morett, and Julio Collado-Vides. Regulondb version 7.0: transcriptional regulation of escherichia coli k-12 integrated within genetic sensory response units (gensor units). *Nucleic Acids Research*, 2010.

[10] Wodek K. Gawronski. *Dynamics and Control of Structures. A Modal Approach.* Mechanical Engineering Series. Springer-Verlag, New York, 1998.

[11] A. M.A. Hamdan and A. H. Nayfeh. Measures of modal controllability and observability for first- and second-order linear systems. *Journal of Guidance, Control, and Dynamics*, 12:421–428, 1989.

[12] C. Josz, S. Fliscounakis, J. Maeght, and P. Panciatici. AC Power Flow Data in MATPOWER and QCQP Format: iTesla, RTE Snapshots, and PEGASE. *ArXiv e-prints*, March 2016.

[13] E.B. Lee and L. Markus. *Foundations of Optimal Control Theory.* R.E. Krieger Publishing Company, 1986.

[14] Haemin Lee and Young-Jin Park. Degree of disturbance rejection capability for linear anti-stable systems. In *Proceedings of the Int. Conf. on Control, Autom., and Syst. (ICCAS)*, 2014.

[15] P. Massa, M. Salvetti, and D. Tomasoni. Bowling alone and trust decline in social network sites. In *2009 Eighth IEEE International Conference on Dependable, Autonomic and Secure Computing*, pages 658–663, Dec 2009.

[16] Peter J. Menck, Jobst Heitzig, Jürgen Kurths, and Hans Joachim Schellnhuber. How dead ends undermine power grid stability. *Nat Commun*, 5, 06 2014.

[17] R. Milo, S. Shen-Orr, S. Itzkovitz, N. Kashtan, D. Chklovskii, and U. Alon. Network motifs: Simple building blocks of complex networks. *Science*, 298(5594):824–827, 2002.

[18] P.C. Müller and H.I. Weber. Analysis and optimization of certain qualities of controllability and observability for linear dynamical systems. *Automatica*, 8(3):237 – 246, 1972.

[19] Kanae Oda, Tomomi Kimura, Yukiko Matsuoka, Akira Funahashi, Masaaki Muramatsu, and Hiroaki Kitano. Molecular interaction map of a macrophage. *AfCS Research Reports*, 2(14 DA), 2004.

[20] Kanae Oda and Hiroaki Kitano. A comprehensive map of the toll-like receptor signaling network. *Mol. Syst. Biol.*, 2:2006.0015, 2006.

[21] Jennifer L. Reed, Thuy D. Vo, Christophe H. Schilling, and Bernhard Ø. Palsson. An expanded genome-scale model of *Escherichia coli* K-12 (*i*JR904 GSM/GPR). *Genome Biol.*, 4(9):R54, 2003.

[22] Hamid Reza Shaker and Maryamsadat Tahavori. Optimal sensor and actuator location for unstable systems. *Journal of Vibration and Control*, 2012.

[23] Eduardo D. Sontag. *Mathematical Control Theory: Deterministic Finite Dimensional Systems (2Nd Ed.)*. Springer-Verlag New York, Inc., New York, NY, USA, 1998.

[24] H. Trentelman, A.A. Stoorvogel, and M. Hautus. *Control Theory for Linear Systems*. Communications and Control Engineering. Springer London, 2012.

[25] R. E. Ulanowicz and D. L. DeAngelis. Network analysis of trophic dynamics in south Forida ecosystems. *US Geological Survey Program on the South Florida Ecosystem*, 114, 2005.

[26] Duncan J. Watts and Steven H. Strogatz. Collective dynamics of small-world networks. *Nature*, 393(6684):440–442, 06 1998.

[27] Kemin Zhou, Gregory Salomon, and Eva Wu. Balanced realization and model reduction for unstable systems. *International Journal of Robust and Nonlinear Control*, 9(3):183–198, 1999.

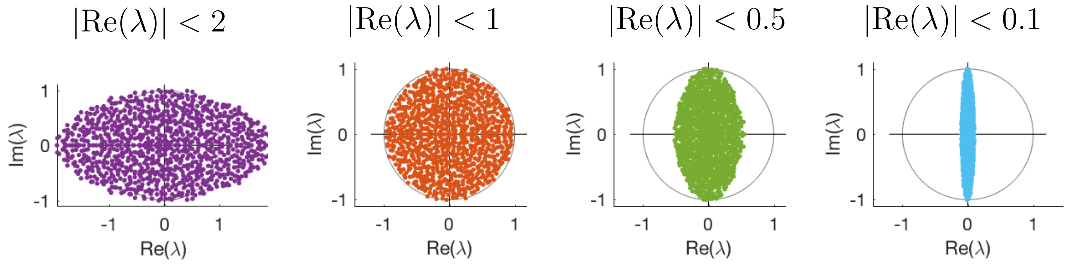

(a)

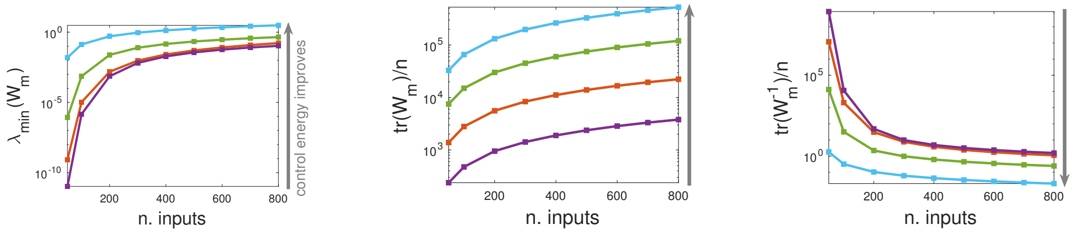

(b)

Figure S1: Analogous of Fig. 2, but for networks with an ER topology with edge probability $p = 0.05$. (a): Four different eigenvalues locations in the complex plane for ER graphs of size $n = 1000$ and random edges weights. The circular law and the elliptic law are still valid. (b): Control energy for various metrics when the number of (randomly chosen) inputs grows. The data show a mean over 100 realizations of dimension $n = 1000$ (for each realization 100 different edge weights assignments are considered). The color code is as in (a). For all three metrics used to measure the control energy ($\lambda_{\min}(W_m)$, $\mathrm{tr}(W_m)$ which should both be maximized, and $\mathrm{tr}(W_m^{-1})$ which should be minimized), the performances are strictly a function of the position of the eigenvalues of A. The minimum of the control energy is achieved when the eigenvalues have very small real part (cyan) and worsen with growing real part, following the order: cyan, green, red, violet.

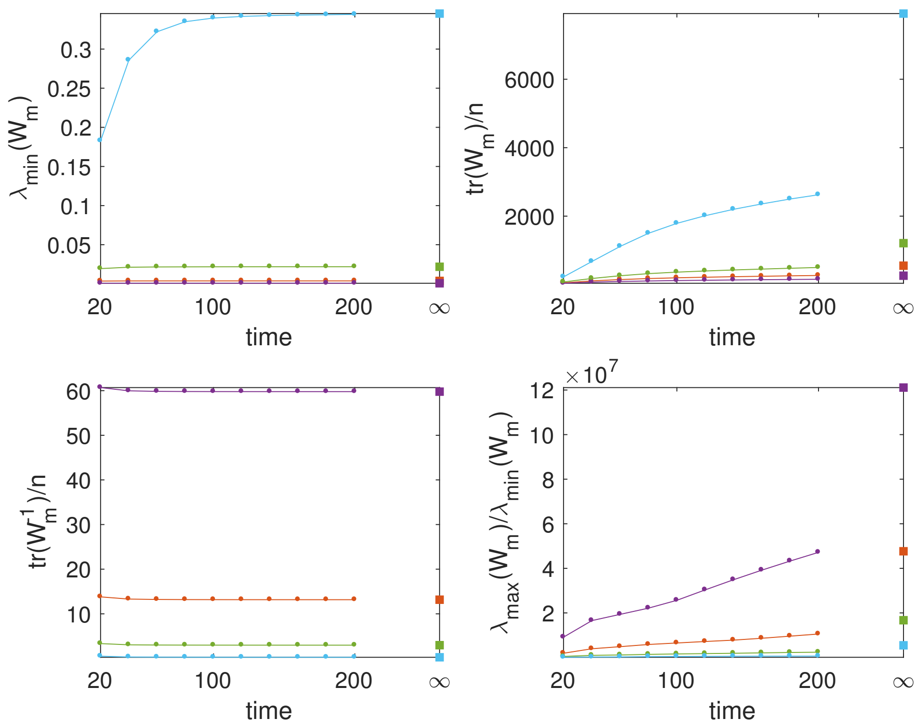

Figure S2: Computing control energies in finite time and infinite time. For the various measures of control energy considered in the paper ($\lambda_{\min}(W_m)$, $\mathrm{tr}(W_m)$ and $\mathrm{tr}(W_m^{-1})$), the plots show the profile in time when computations are performed using $W_m(t_f)$, for various values of t_f. The value for $t_f = \infty$ is also shown for comparison. For this specific example (full network of size $n = 1000$ and $m = 400$ controls), some measures converge much faster than others. For instance $\mathrm{tr}(W_m^{-1})$ achieves its infinite-time value extremely quickly, while $\mathrm{tr}(W_m)$ converges very slowly. Also the condition number of W_m (i.e., $\lambda_{\max}(W_m)/\lambda_{\min}(W_m)$, lower right panel) converges to its asymptotic value.

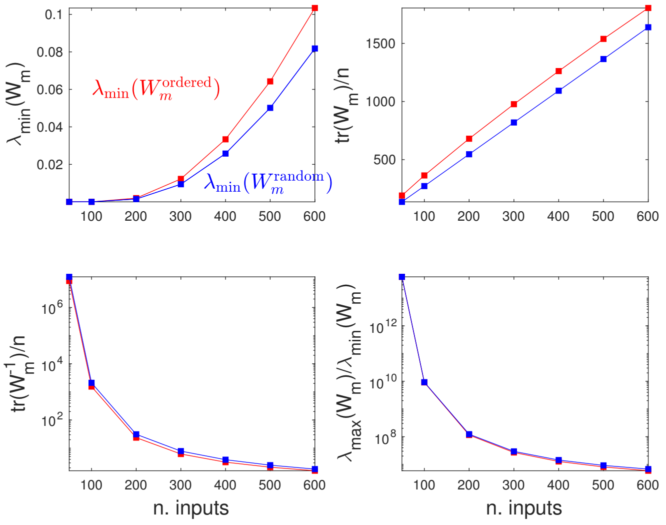

(a)

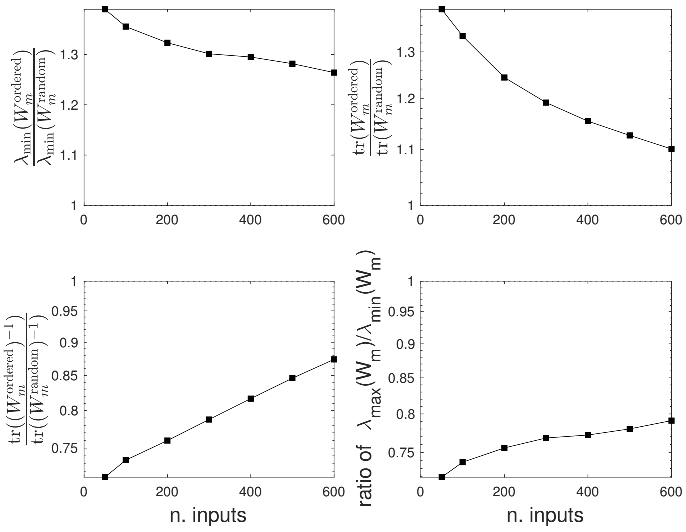

(b)

Figure S3: Driver node placement for ER networks with $p = 0.05$. (a): Comparison between the value of the various measures of control energy obtained for driver node placement strategies based on $r_w = w_{\text{out}}/w_{\text{in}}$ (red, labelled "ordered") and the same measure for random driver node assignments (blue, labelled "random"). As can be seen on the ratios shown in (b), all measures improve, especially when m is low. Notice that also the condition number of W_m (i.e., $\lambda_{\max}(W_m)/\lambda_{\min}(W_m)$, lower right panel) improves.

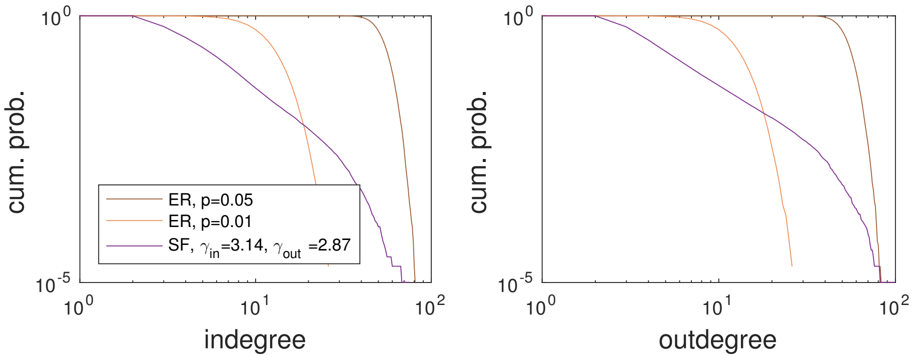

(a)

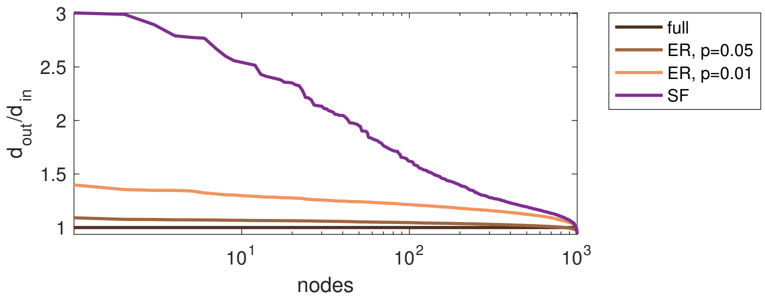

(b)

Figure S4: Degree distributions of the ER and SF networks used in this study. (a): Average indegree and outdegree of the ER networks (with $p = 0.05$ and $p = 0.01$) and SF networks. The SF networks are generated using the algorithm of [4], with indegree exponent $\gamma_{\rm in} = 3.14$ and outdegree exponent $\gamma_{\rm out} = 2.87$. To avoid problems with controllability, extra edges are added randomly among different strongly connected components, until strong connectivity is achieved on the entire graph. The difference in the in/out exponent is still clearly visible. (b): Histogram of outdegree/indegree ratio for the various networks. The SF networks reflect our choice of $\gamma_{\rm out} < \gamma_{\rm in}$.

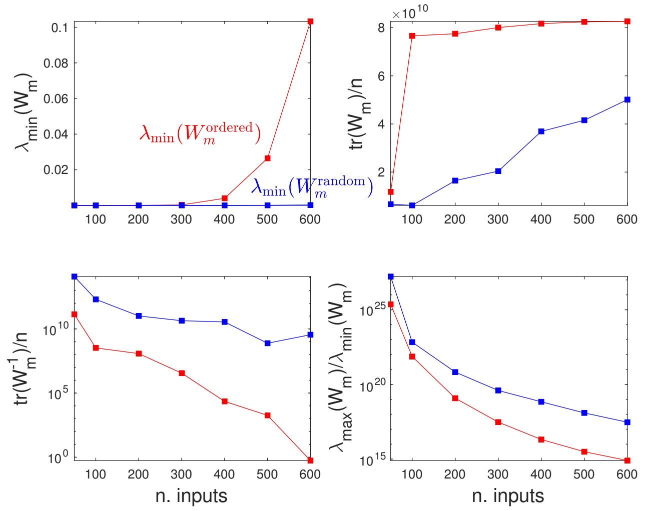

(a)

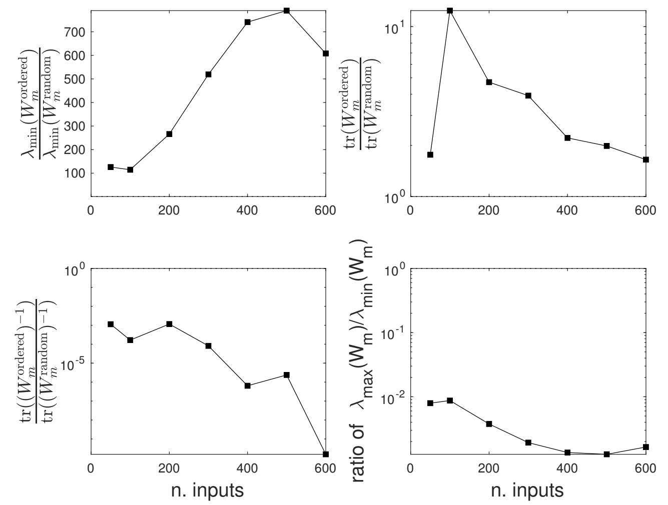

(b)

Figure S5: Driver node placement for SF networks with $\gamma_{\text{in}} = 3.14$ and $\gamma_{\text{out}} = 2.87$. (a): Comparison between the value of the various measures of control energy obtained for driver node placement strategies based on $r_w = w_{\text{out}}/w_{\text{in}}$ (red, labelled "ordered") and the same measure for random driver node assignments (blue, labelled "random"). As the ratios in (b) show, the improvement in all measures is normally of several orders of magnitude. Also the condition number of W_m (i.e., $\lambda_{\max}(W_m)/\lambda_{\min}(W_m)$) improves substantially.

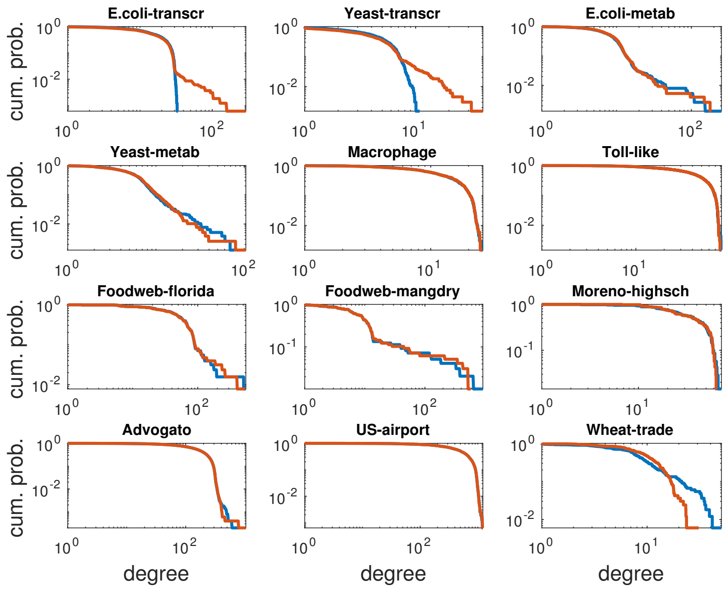

(a)

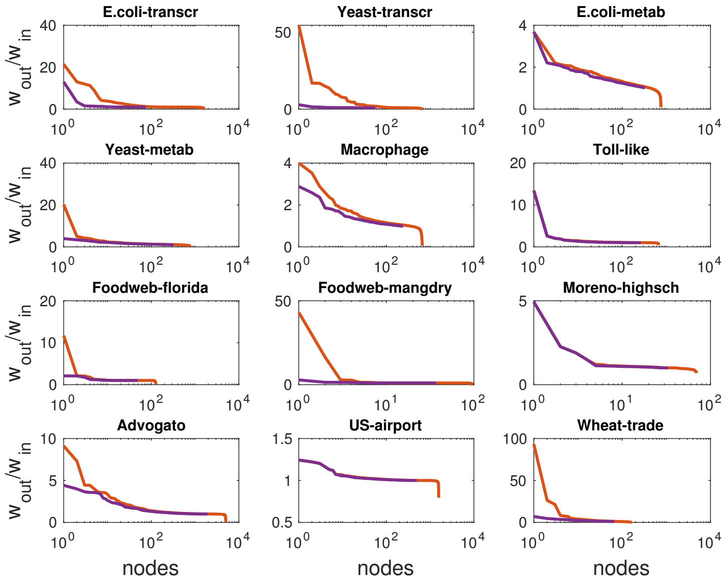

(b)

Figure S6: Degree distributions and r_w rankings of the real-world networks of Table 1 of the paper. (a): Degree distributions (indegree in blue and outdegree in red). (b): Ranked ratio $r_w = w_{\text{out}}/w_{\text{in}}$ for the same networks, in red for the whole network, in violet only for the extra m_e nodes selected as driver nodes (in addition to the m_c nodes needed for controllability).

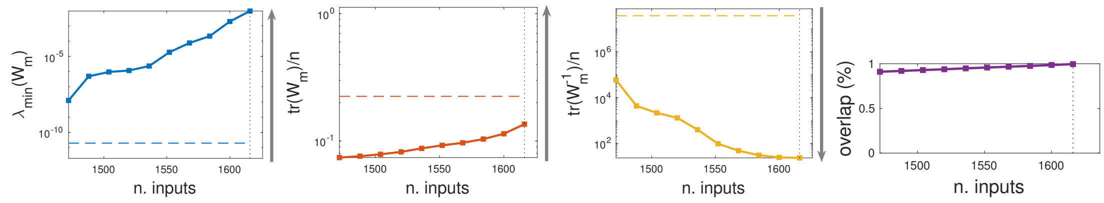

(a) *E.coli-transcr*

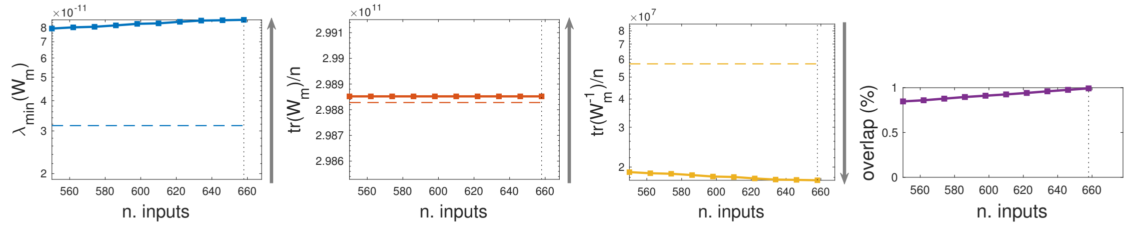

(b) *Yeast-transcr*

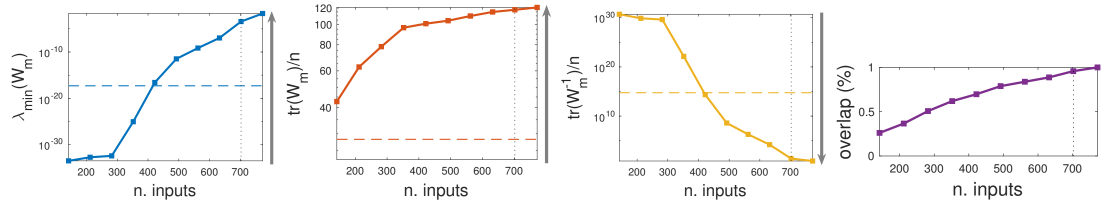

(c) *Yeast-metab*

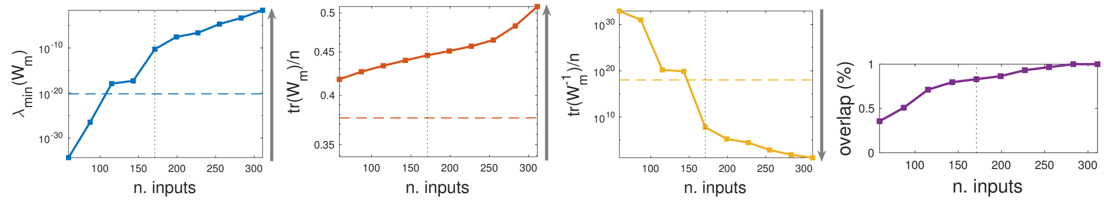

(d) *Egfr*

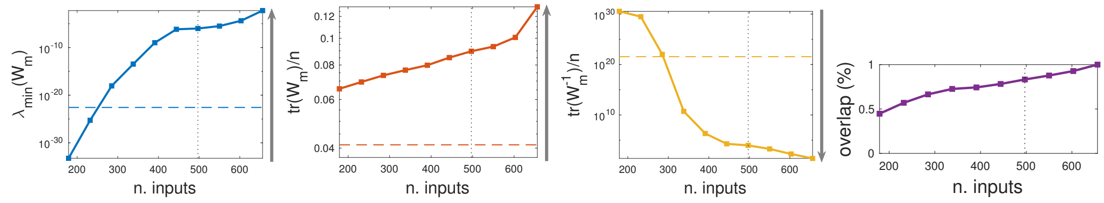

(e) *Macrophage*

Figure S7: Driver node placement according to $r_w = w_{\text{out}}/w_{\text{in}}$ real-world networks of Table 1 of the paper (part 1). In all subfigures the control energy measures $\lambda_{\min}(W_m)$, $\text{tr}(W_m)$, and $\text{tr}(W_z^{-1})$ are shown in the 3 leftmost panels (solid lines), when the number m_e of driver nodes (chosen according to r_w) grows. The horizontal dashed line is the control energy in correspondence of the m_c driver nodes required by structural controllability. The vertical dotted line is the value of m_e at which the r_w-ranked nodes achieve structural controllability. The rightmost panel shows how many of the m_e r_w-ranked nodes overlap with the m_c nodes of structural controllability.

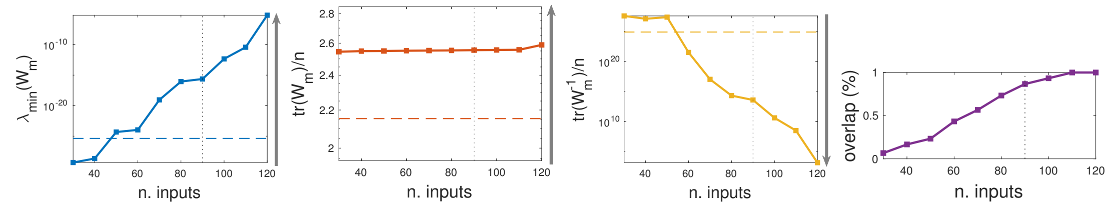

(a) *Foodweb-florida*

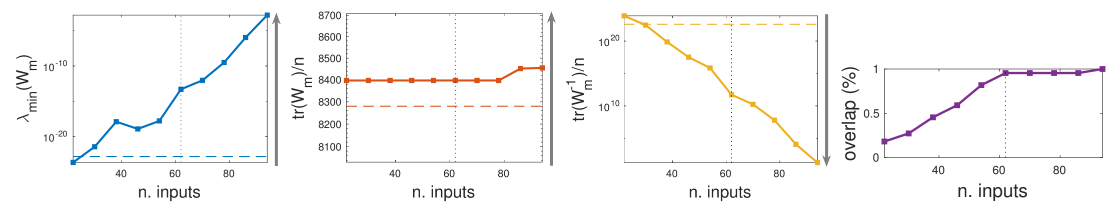

(b) *Foodweb-magdry*

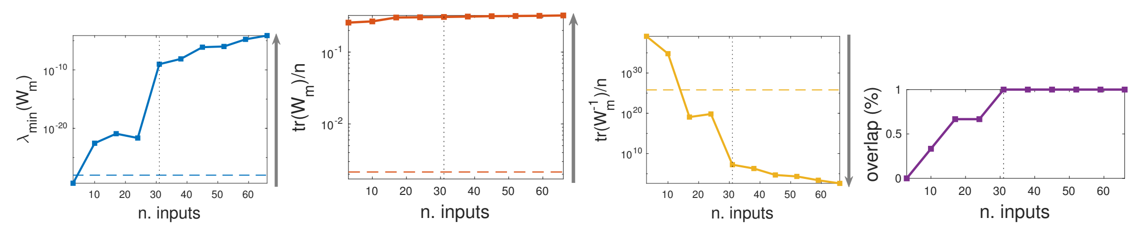

(c) *Moreno*

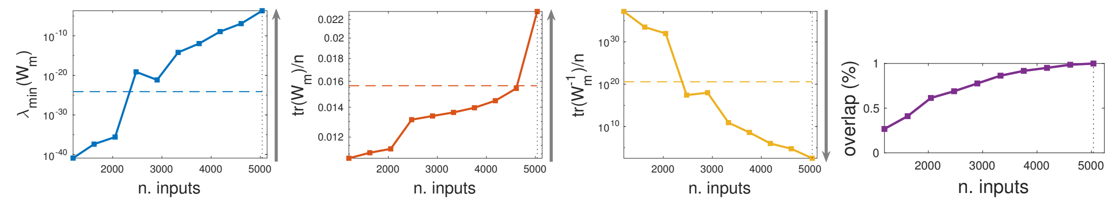

(d) *Advogato*

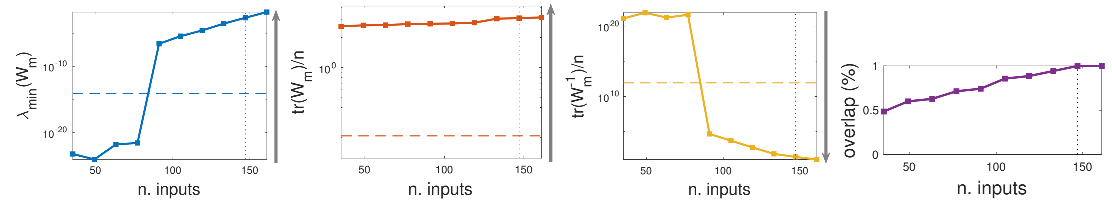

(e) *Wheat-trade*

Figure S8: Driver node placement according to $r_w = w_{\rm out}/w_{\rm in}$ real-world networks of Table 1 of the paper (part 2). In all subfigures the control energy measures $\lambda_{\min}(W_m)$, $\mathrm{tr}(W_m)$, and $\mathrm{tr}(W_z^{-1})$ are shown in the 3 leftmost panels (solid lines), when the number m_e of driver nodes (chosen according to r_w) grows. The horizontal dashed line is the control energy in correspondence of the m_c driver nodes required by structural controllability. The vertical dotted line is the value of m_e at which the r_w-ranked nodes achieve structural controllability. The rightmost panel shows how many of the m_e r_w-ranked nodes overlap with the m_c nodes of structural controllability.

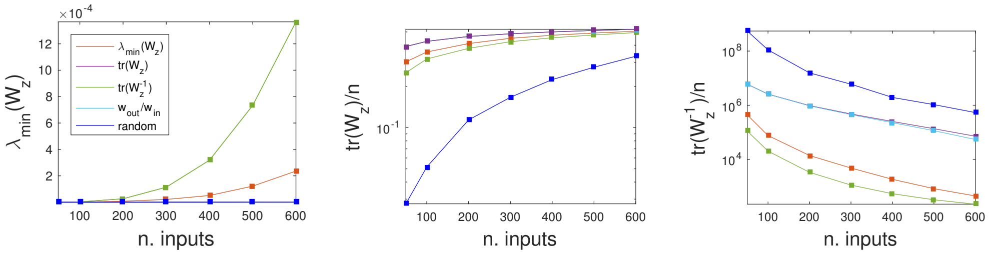

(a)

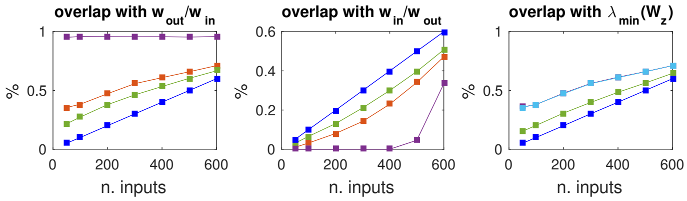

(b)

Figure S9: Driver node placement for a network of $n = 1000$ coupled harmonic oscillators. The figure is the analogous of Fig. 4, but now the coupling matrix K is fully connected. (a): Shown are means over 50 realizations (with 100 edge weight samples taken for each realization). Red: driver node placement based on $\lambda_{\min}(W_z)$. Violet: placement based on $\mathrm{tr}(W_z)$. Green: placement based on $\mathrm{tr}(W_z^{-1})$. Cyan: placement based on $w_{\mathrm{out}}/w_{\mathrm{in}}$. Blue: random input assignment. All driver node placement strategies still beat a random assignment, but with worse performances with respect to Fig. 4. Of the four measures, $\lambda_{\min}(W_z)$ and $\mathrm{tr}(W_z^{-1})$ tend to behave similarly and so do $w_{\mathrm{out}}/w_{\mathrm{in}}$ and $\mathrm{tr}(W_z)$ (in the mid plot they completely overlap, and both give the true optimum). (b): Overlap in the node ranking of the different driver node placement strategies. Color code is the same as in (a). The only highly significant overlap is still between $w_{\mathrm{out}}/w_{\mathrm{in}}$ and $\mathrm{tr}(W_z)$ ($> 90\%$), while $\lambda_{\min}(W_z)$ and $\mathrm{tr}(W_z^{-1})$ correspond to different node ranking patterns. None of the strategies orders nodes according to $w_{\mathrm{in}}/w_{\mathrm{out}}$, as expected.

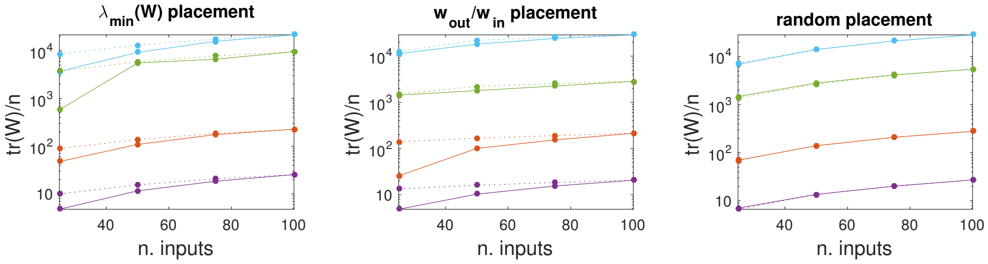

(a)

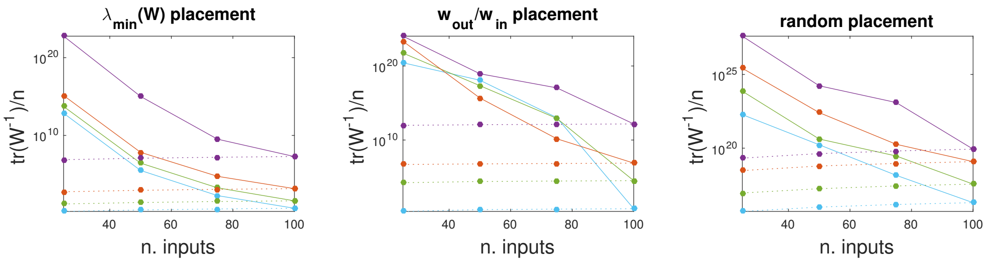

(b)

Figure S10: Minimum energy control of power grids with varying damping coefficients. North EU power grid. This Figure complements Fig. 5(b) of the paper. (a): Control energy for the metric $\mathrm{tr}(W_r)$ when the driver nodes are placed according to $\lambda_{\min}(W_r)$ (left panel), $w_{\mathrm{out}}/w_{\mathrm{in}}$ (mid panel), or randomly (right panel). The color code is a function of the damping coefficients, with the same convention as in Fig. 5(a) of the paper. The values of $\mathrm{tr}(W_r)$ are shown in solid lines, while in dotted lines the values of $\mathrm{tr}(W_z)$ are shown (suitably normalized to eliminate the explicit dependence from t_f). Values are averages over 100 realizations. (b): Control energy for the metric $\mathrm{tr}(W_r^{-1})$, with the same conventions as in (a).

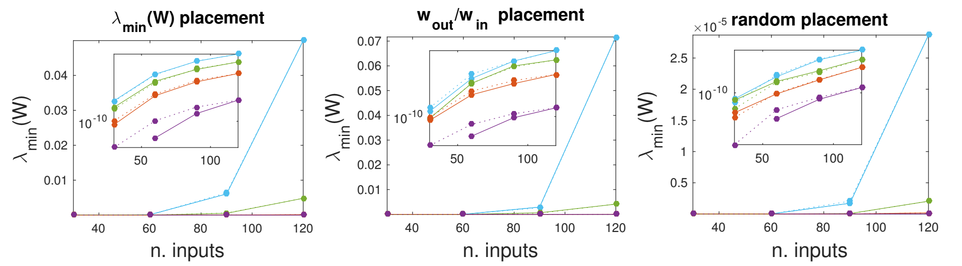

(a)

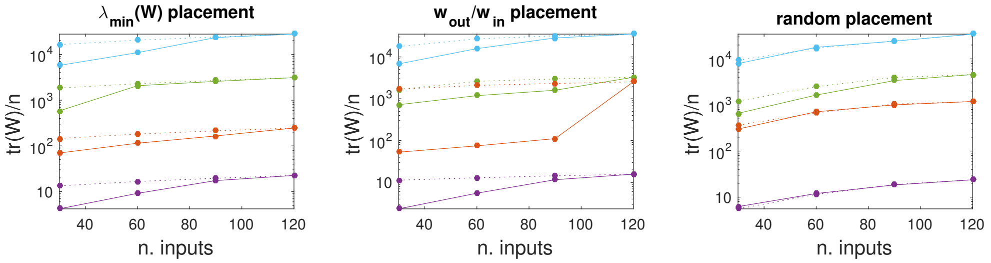

(b)

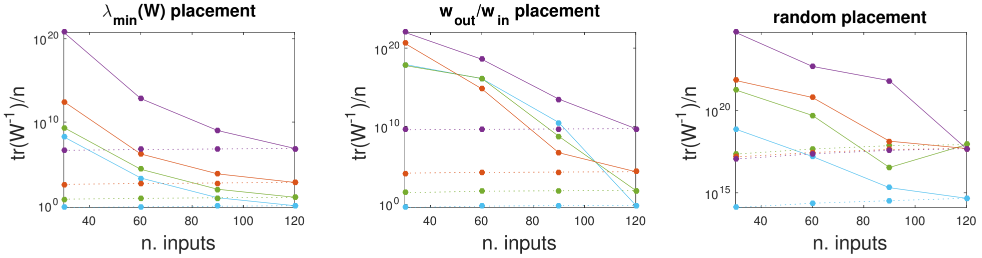

(c)

Figure S11: Minimum energy control of power grids with varying damping coefficients. IEEE 300 bus test power network. (a): Control energy for the metric $\lambda_{\min}(W_r)$ when the driver nodes are placed according to $\lambda_{\min}(W_r)$ (left panel), $w_{\text{out}}/w_{\text{in}}$ (mid panel), or randomly (right panel). The color code is a function of the damping coefficients, using the same convention as in Fig. 5(a) of the paper. The values of $\lambda_{\min}(W_r)$ are shown in solid lines, while in dotted lines the values of $\lambda_{\min}(W_z)$ are shown (suitably normalized to eliminate the explicit dependence from t_f). Values are averages over 100 realizations. Data are missing when the Gramian W_r is too close to singular in too many trials. (b): Control energy for the metric $\text{tr}(W_r)$ when the driver nodes are placed according to $\lambda_{\min}(W_r)$ (left panel), $w_{\text{out}}/w_{\text{in}}$ (mid panel), or randomly (right panel). The values of $\text{tr}(W_r)$ are shown in solid lines, while in dotted lines the values of $\text{tr}(W_z)$ are shown. (c): Control energy for the metric $\text{tr}(W_r^{-1})$, with the same conventions as in (a) and (b).

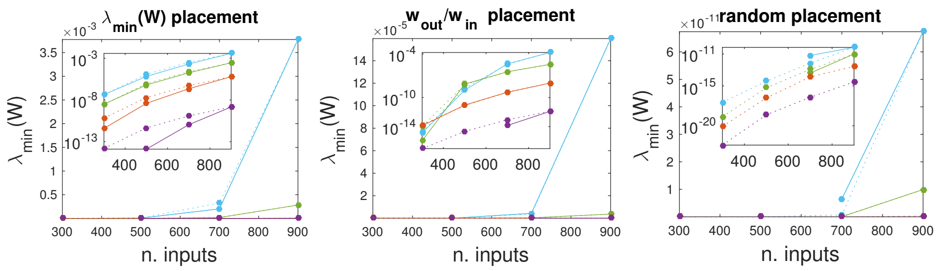

(a)

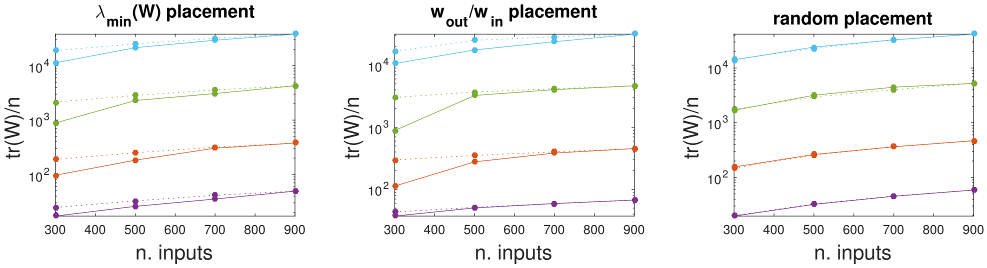

(b)

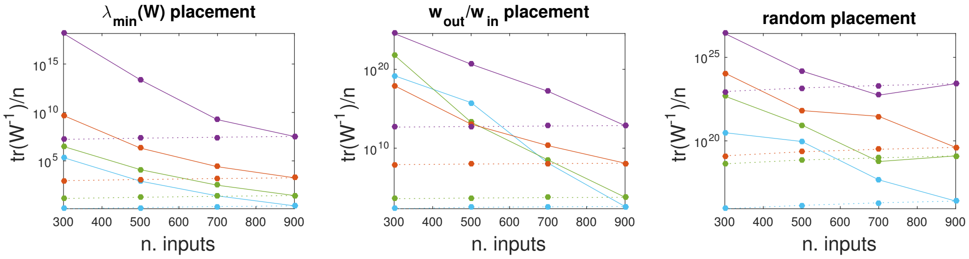

(c)

Figure S12: Minimum energy control of power grids with varying damping coefficients. French high/mid voltage power grid. (a): Control energy for the metric $\lambda_{\min}(W_r)$ when the driver nodes are placed according to $\lambda_{\min}(W_r)$ (left panel), $w_{\text{out}}/w_{\text{in}}$ (mid panel), or randomly (right panel). The color code is a function of the damping coefficients, using the same convention as in Fig. 5(a) of the paper. The values of $\lambda_{\min}(W_r)$ are shown in solid lines, while in dotted lines the values of $\lambda_{\min}(W_z)$ are shown (suitably normalized to eliminate the explicit dependence from t_f). Data are missing when the Gramian W_r is too close to singular (mostly when driver nodes are chosen randomly, right column). (b): Control energy for the metric $\text{tr}(W_r)$ when the driver nodes are placed according to $\lambda_{\min}(W_r)$ (left panel), $w_{\text{out}}/w_{\text{in}}$ (mid panel), or randomly (right panel). The values of $\text{tr}(W_r)$ are shown in solid lines, while in dotted lines the values of $\text{tr}(W_z)$ are shown. (c): Control energy for the metric $\text{tr}(W_r^{-1})$, with the same conventions as in (a) and (b).

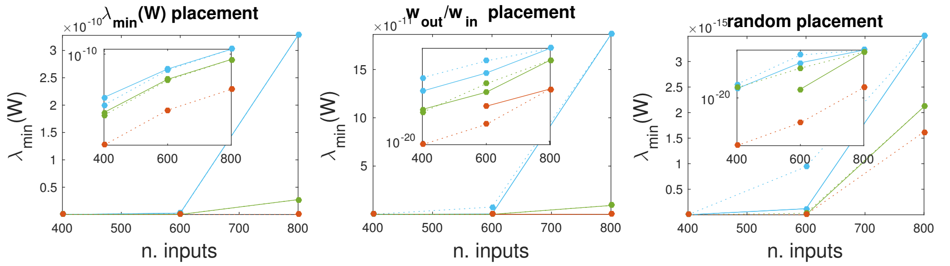

(a)

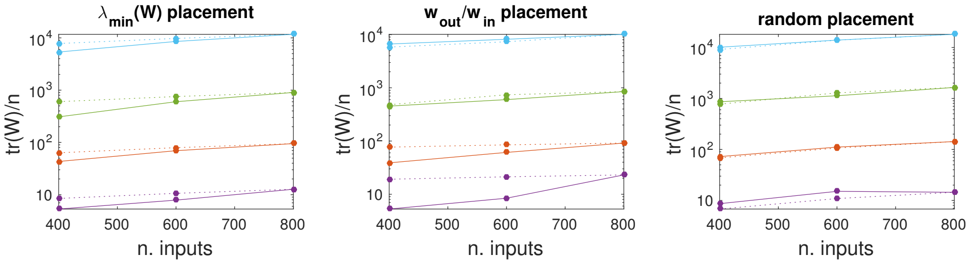

(b)

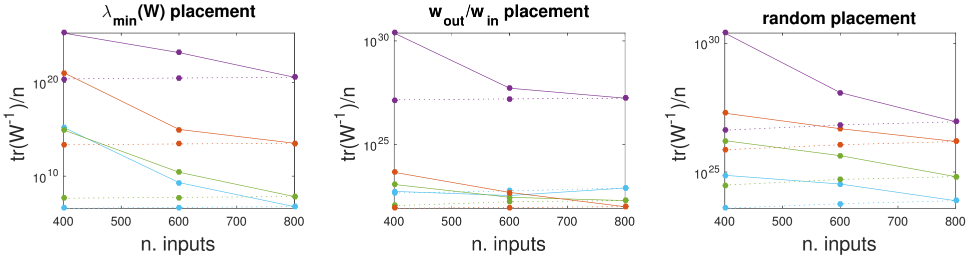

(c)

Figure S13: Minimum energy control of power grids with varying damping coefficients. USA power grid. (a): Control energy for the metric $\lambda_{\min}(W_r)$ when the driver nodes are placed according to $\lambda_{\min}(W_r)$ (left panel), $w_{\text{out}}/w_{\text{in}}$ (mid panel), or randomly (right panel). The color code is a function of the damping coefficients, using the same convention as in Fig. 5(a) of the paper. The values of $\lambda_{\min}(W_r)$ are shown in solid lines, while in dotted lines the values of $\lambda_{\min}(W_z)$ are shown (suitably normalized to eliminate the explicit dependence from t_f). Data are missing when the Gramian W_r is numerically too close to singular in too many trials. (b): Control energy for the metric $\text{tr}(W_r)$ when the driver nodes are placed according to $\lambda_{\min}(W_r)$ (left panel), $w_{\text{out}}/w_{\text{in}}$ (mid panel), or randomly (right panel). The values of $\text{tr}(W_r)$ are shown in solid lines, while in dotted lines the values of $\text{tr}(W_z)$ are shown. (c): Control energy for the metric $\text{tr}(W_r^{-1})$, with the same conventions as in (a) and (b).
